# YAP1 Exerts Its Transcriptional Control via TEAD-Mediated Activation of Enhancers

**DOI:** 10.1371/journal.pgen.1005465

**Published:** 2015-08-21

**Authors:** Claudia Stein, Anaïs Flore Bardet, Guglielmo Roma, Sebastian Bergling, Ieuan Clay, Alexandra Ruchti, Claudia Agarinis, Tobias Schmelzle, Tewis Bouwmeester, Dirk Schübeler, Andreas Bauer

**Affiliations:** 1 Developmental and Molecular Pathways, Novartis Institutes for Biomedical Research, Novartis Pharma AG, Basel, Switzerland; 2 Friedrich Miescher Institute for Biomedical Research, Basel, Switzerland; 3 Oncology, Novartis Institutes for Biomedical Research, Novartis Pharma AG, Basel, Switzerland; 4 University of Basel, Faculty of Sciences, Basel, Switzerland; The University of North Carolina at Chapel Hill, UNITED STATES

## Abstract

YAP1 is a major effector of the Hippo pathway and a well-established oncogene. Elevated YAP1 activity due to mutations in Hippo pathway components or *YAP1* amplification is observed in several types of human cancers. Here we investigated its genomic binding landscape in YAP1-activated cancer cells, as well as in non-transformed cells. We demonstrate that TEAD transcription factors mediate YAP1 chromatin-binding genome-wide, further explaining their dominant role as primary mediators of YAP1-transcriptional activity. Moreover, we show that YAP1 largely exerts its transcriptional control via distal enhancers that are marked by H3K27 acetylation and that YAP1 is necessary for this chromatin mark at bound enhancers and the activity of the associated genes. This work establishes YAP1-mediated transcriptional regulation at distal enhancers and provides an expanded set of target genes resulting in a fundamental source to study YAP1 function in a normal and cancer setting.

## Author Summary

The YAP1/Hippo signaling pathway is a key regulator of organ size and tissue homeostasis, and its dysregulation is linked to cancer development. Elevated activity of YAP1, a transcriptional coactivator and well-established oncogene has been reported to occur in human cancers. Comprehensive identification of YAP1 regulated genes and its mode of action will be of high importance to uncover YAP1 biology that could be exploited for a therapeutic intervention. To this end, we performed genome-wide analyses to identify YAP1 occupied sites in cancer cell lines representing different YAP1/Hippo pathway tumor etiologies and in non-transformed fibroblasts. Our data demonstrate that YAP1 activity is mediated predominantly via TEAD transcription factors supporting the importance of TEADs as main mediators of YAP1-coactivator activity. We further show that YAP1 and TEAD1 exert their transcriptional control via binding to enhancers, leading to characteristic chromatin changes and distal activation of genes. By linking enhancers to genes, we provide a list of novel YAP1 target genes in an oncogenic setting that we show can readily be exploited in tumor classification and provides a foundation for further investigations.

## Introduction

YAP1 (Yes-associated protein 1) is a major transcriptional effector of the evolutionary and functionally conserved Hippo pathway, which is a crucial regulator of organ size, proliferation but also tumor growth [1–3]. Activation of the Hippo pathway leads to phosphorylation and inactivation of the transcriptional co-activator YAP1 by cytoplasmic retention or enhanced degradation [4–8].

YAP1 has a potent growth promoting activity and the YAP1/Hippo pathway has been tightly linked to cancer [8–11]. Loss of Hippo signaling by mutations or down-regulation of core pathway components is associated with cancer development, while YAP1 is reported as a potent oncogene that can promote tumorigenesis in a wide range of tissues [2, 12, 13]. Elevated expression or activity of YAP1 occurs through multiple mechanisms. *YAP1* gene amplification and mutations in upstream pathway regulators, such as *NF2*, have been described in various human tumors [2, 14–20].

YAP1 lacks an intrinsic DNA-binding domain and is thought to exert its co-activator function through binding to promoter sequences via interaction with transcription factors (TF), such as TEAD1/-2/-3/-4, Smads, Runx1/-2, p73, ErbB4, Pax3, AP-1, or TBX5 [12, 21]. Among these the TEAD TF family members play a dominant role as primary mediators of YAP1-dependent gene regulation and YAP1 growth-promoting activity [22–28]. Although the tumor-promoting function of YAP1 and TEAD by controlling a remarkable range of cellular processes is undisputed [1, 13, 27], the comprehensive ensemble of direct downstream target genes and the underlying mechanisms of target gene regulation remain poorly understood.

In the past decade, gene expression studies have identified several YAP1-responsive genes [22, 29–31]. In contrast, the number of validated direct target genes remains small. Besides validating YAP1 binding to proximal promoter regions of individual genes using ChIP-qPCR [22, 29, 31–38], a ChIP-on-chip approach using a microarray consisting of promoter regions has been conducted to identify direct YAP1-target genes in MCF10A mammary epithelial cells [22]. While focusing on YAP1-binding to promoter proximal regions a substantial set of functional YAP1 genomic binding sites might have been missed given the importance of distal regulatory elements in establishing a precise pattern of gene expression [39–43].

Here, we comprehensively mapped YAP1 chromatin binding genome-wide, independent of gene location, using ChIP-seq in two human cancer cell lines from different lineages with elevated YAP1 activity (SF268 and NCI-H2052) as well as in non-transformed cells (IMR90) enabling an unbiased identification of YAP1 binding sites and their dependence on cellular context. We demonstrate that YAP1 chromatin recruitment is primarily mediated by binding of TEAD1 to single as well as double TEAD motifs with 3bp spacer at distal enhancers. Aside from presenting a global view of YAP1 and TEAD1 binding in a cancer context, our study also provides novel mechanistic insights into YAP1 transcriptional co-activation of TEAD TFs. We show that YAP1-dependent enhancer activation entails characteristic chromatin changes at lysine 27 of histone H3 and activation of associated genes. Finally we identify a set of YAP1 targets genes by expression profiling following YAP1 knockdown representing a gene signature that can predict YAP1 activity in tumor samples.

## Results

### Genome-wide YAP1 chromatin-binding in *YAP1*-amplified cancer cells

**Fig 1:**
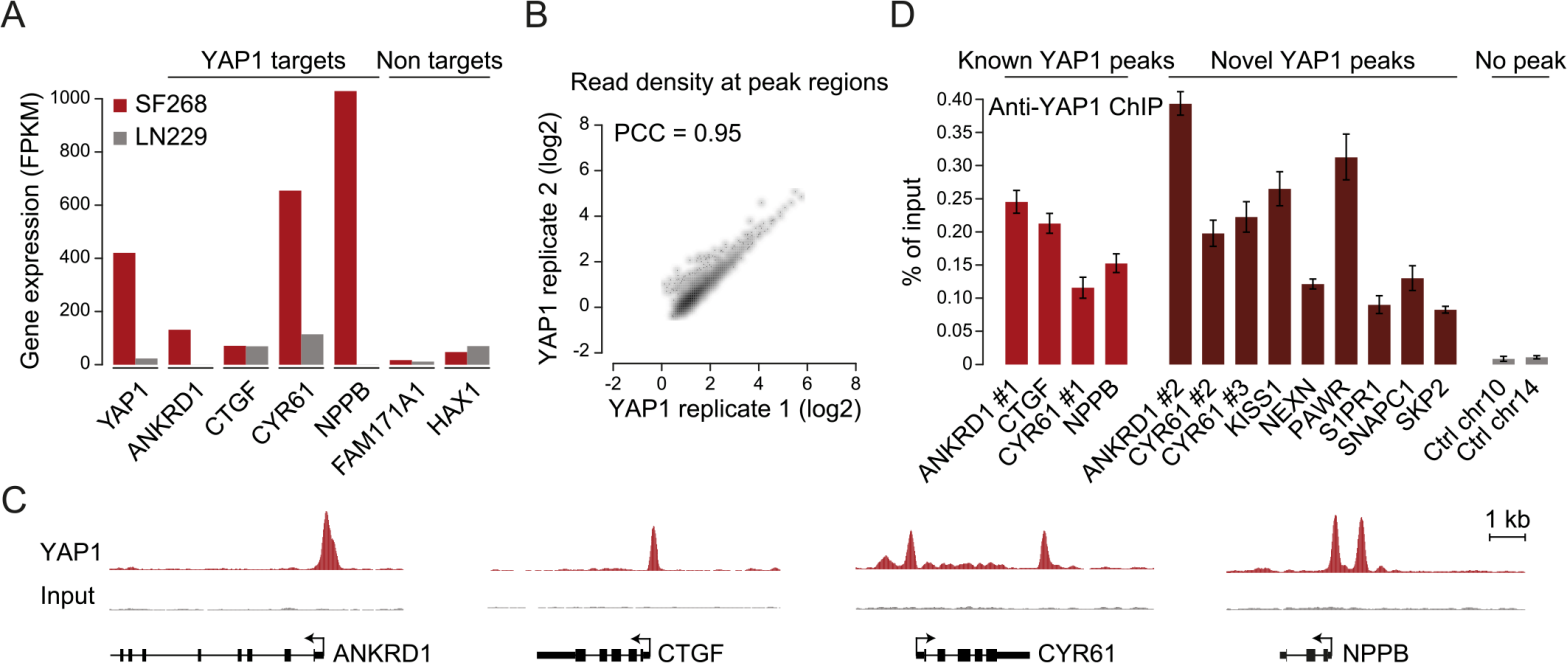
Genome-wide binding of YAP1 to chromatin in SF268 cells. (A) Expression levels of *YAP1*, YAP1 target genes and non-target genes in SF268 (red) and LN229 (grey) cells measured by RNA-seq. (B) Correlation between replicates of YAP1 binding analysis by ChIP-seq. (C) Genomic views of YAP1 ChIP enrichment at gene promoters of known target genes. (D) Validation of YAP1 binding to known and novel sites, and control regions using ChIP-qPCR. Data are representative of at least three independent experiments. Error bars indicate the standard deviation of triplicate qPCR.

To gain insight into YAP1 genomic recruitment in a YAP1-relevant cancer context, we used SF268 glioblastoma cells, previously demonstrated to have elevated YAP1 activity due to a 13-fold genomic amplification of the *YAP1* locus [44]. Accordingly, YAP1 mRNA and protein levels are increased in SF268 cells as compared to LN229 glioblastoma cells that do not harbor any genetic aberrations of YAP1/Hippo pathway components (Fig 1A and S1 Fig). As a consequence, YAP1 transcriptional activity appears significantly elevated, as suggested by an increased expression of known YAP1 target genes *ANKRD1*, *CYR61*, *and NPPB* but not of unrelated genes *FAM171A1* and *HAX1* (Fig 1A).

To identify YAP1 binding sites genome-wide we performed chromatin immunoprecipitation with a YAP1-specific antibody followed by high-throughput sequencing (ChIP-seq). The chosen antibody proved to be highly specific and sensitive as measured by western blot analysis as well as immunoprecipitation (S2 Fig). We observed high reproducibility between two independent biological ChIP-seq replicates with a Pearson correlation coefficient (PCC) of 0.95 (Fig 1B). We identified 2,498 binding sites enriched over matching input using the ChIP-seq peak-finder peakzilla [45] (S1 and S2 Tables). To further benchmark our approach we have analyzed the dataset for the presence of peak regions in the most commonly described YAP1 target genes. As anticipated, peaks were identified in the vicinity of published YAP1 target genes, such as *CTGF* [22], *CYR61* [6], *NPPB* [32], *CCND1* [31], *AXL* [36], *DKK1* [33], *ITGB2* [22], *WWC1* [35], *and ANKRD1* (Fig 1C and S3 Fig). Although *ANKRD1* expression is commonly used to monitor YAP1 transcriptional activity, to our knowledge, it has not formerly been proven as a direct YAP1 target gene. Our data proofs direct YAP1 binding to the promoter of *ANKRD1* (Fig 1C and 1D). ChIP-qPCR validation for several randomly selected loci confirmed YAP1 occupancy at those sites (Fig 1D), further supporting the specificity of binding and overall reliability of the dataset.

### The TEAD consensus motif is enriched in YAP1 binding sites

**Fig 2:**
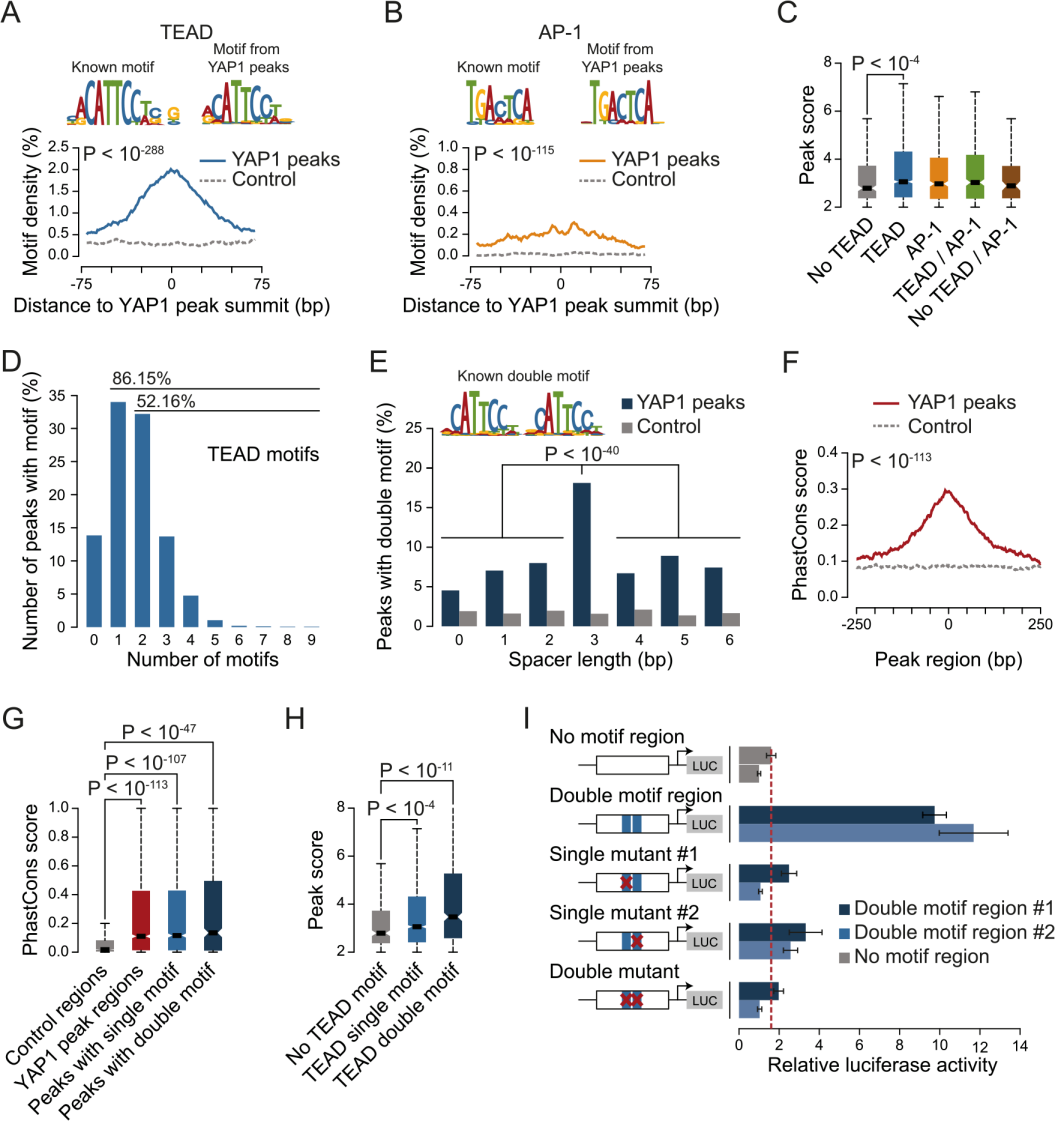
TEAD single and double motifs occur within most YAP1 binding sites. (A and B) Enrichment of (A) TEAD and (B) AP-1 motifs in YAP1 peaks. Full list provided in S4 Table. (C) YAP1 ChIP enrichment as determined by peak score in YAP1 peaks with/without TEAD and AP-1 motifs. (D) Number of TEAD motifs in YAP1 peaks. (E) Enrichment of TEAD double motif with several spacer lengths in YAP1 peaks. (F) Sequence conservation of YAP1 peak regions. (G) Sequence conservation of TEAD single and double motifs in YAP1 peak regions. (H) YAP1 ChIP enrichment as determined by peak score in YAP1 peaks with/without single/double TEAD motifs. (I) Luciferase reporter assay for two YAP1 binding regions with either intact double motif or with single or double mutations. Relative luciferase activity represents the ratio of Firefly and Renilla luciferase activity for each sample. The red line indicates the highest mean activity of the two negative control regions. Data are representative of at least three independent experiments. Error bars indicate the standard deviation of triplicate qPCR data.

YAP1 does not contain a DNA-binding domain and thus, relies on interactions with other TFs for recruitment to chromatin. To investigate which TFs mediate binding in SF268 cells we searched the YAP1 peak regions for motifs *de novo* using MEME [46]. This identified *CATTCC*, the known consensus motif for TEAD, as the predominant hit (Fig 2A). When allowing for 1 base pair (bp) mismatches to the TEAD consensus motif (S3 Table) we observed that more than 86% of all YAP1 peak regions contained at least one TEAD binding site. This represents a 2.3-fold enrichment over random control regions (hypergeometric *P* < 10^−288^) and provides evidence that TEADs are the predominant co-factors facilitating YAP1 association with chromatin in *YAP1*-amplified glioblastoma cancer cells.

To ask whether additional TFs might recruit YAP1, we searched YAP1 peak regions for enrichment of other known TF motifs. Besides the TEAD consensus motif, we identified only the AP-1/JDP2 motif *TGACTCA* to be significantly enriched (Fig 2B and S4 and S5 Tables). AP-1 is a heterodimeric protein complex composed of c-Fos and c-Jun, both highly expressed in SF268 cells (S5 Table). Cooperative binding of AP-1 with other TFs has been previously reported as a mechanism of context specific gene regulation [47]. Therefore YAP1/TEAD might act cooperatively with AP-1 in a stimulation-dependent manner or dependent on the pathway genetic context of the analyzed cell type to regulate context-specific gene expression programs. In support of this, c-Fos has recently been described to regulate YAP1 transcriptional activity in the context of *KRAS*-driven cancers [30] and AP-1/TEAD were found to act as regulators of the invasive gene network in melanoma [48]. When allowing for 1bp mismatches we identified *TGACTCA* motifs in 60% of YAP1 peak regions that do not contain a TEAD motif but observed the motif as well in 45% of peak regions with a TEAD binding motif. Furthermore we observe that peak regions containing a TEAD binding motif have significantly higher YAP1 ChIP occupancy (as defined by the peakzilla peak score), while the presence of an AP-1 motif does not significantly increase YAP1 occupancy (Fig 2C). Our genome-wide binding data therefore do not provide convincing evidence that AP-1 might serve as an alternative factor for the recruitment of YAP1 to chromatin. However we cannot exclude that AP-1 might serve as a co-factor for YAP1/TEAD under specific experimental conditions.

Taken together, our genome-wide binding data support the notion that TEADs account for the vast majority of YAP1 binding to chromatin.

### A double TEAD motif with a 3bp spacer is enriched and functional in YAP1 binding sites

We noted that 52% of peak regions contained more than one TEAD binding motif (Fig 2D) with two consecutive sites (double motif) being particularly prevalent. Binding of TEADs and other TFs to double motifs has been recently shown *in vitro* using high-throughput SELEX [49]. Indeed, we found a specific enrichment of double motifs oriented in the same direction separated by a 3bp spacer (18%) as compared to other spacing or random control regions (hypergeometric P <10^−145^ vs. control and P < 10^−40^ vs. other spacer lengths) (Fig 2E). This is consistent with a cooperative mechanism of TEAD1 binding to DNA that has previously been suggested based on structural analyses [50] and *in vitro* binding experiments [51, 52].

We observed that peak regions are significantly conserved as compared to random control regions especially at their peak summit (Fig 2F and 2G). Further, in contrast to peaks without a TEAD motif, peaks with single or double motifs had significantly higher ChIP occupancies (Fig 2H).

To directly investigate the functionality of the TEAD double motif we utilized a luciferase reporter gene assay. Double motifs from two independent peak regions (CATTCC-NNN-CATTCC) were cloned upstream of a luciferase reporter. Both constructs caused an increase in luciferase reporter expression as compared to control regions. Importantly, mutations in either one or both of the double motif sites reduced reporter gene expression to the levels of control regions (Fig 2I, red line), indicating that both sites of the double motif are required to enhance transcription.

We conclude that, at a subset of binding sites, TEAD binds homotypic clusters of motifs as previously shown for other human TFs [53].

### YAP1 binding sites are co-occupied by TEAD1 genome-wide

**Fig 3:**
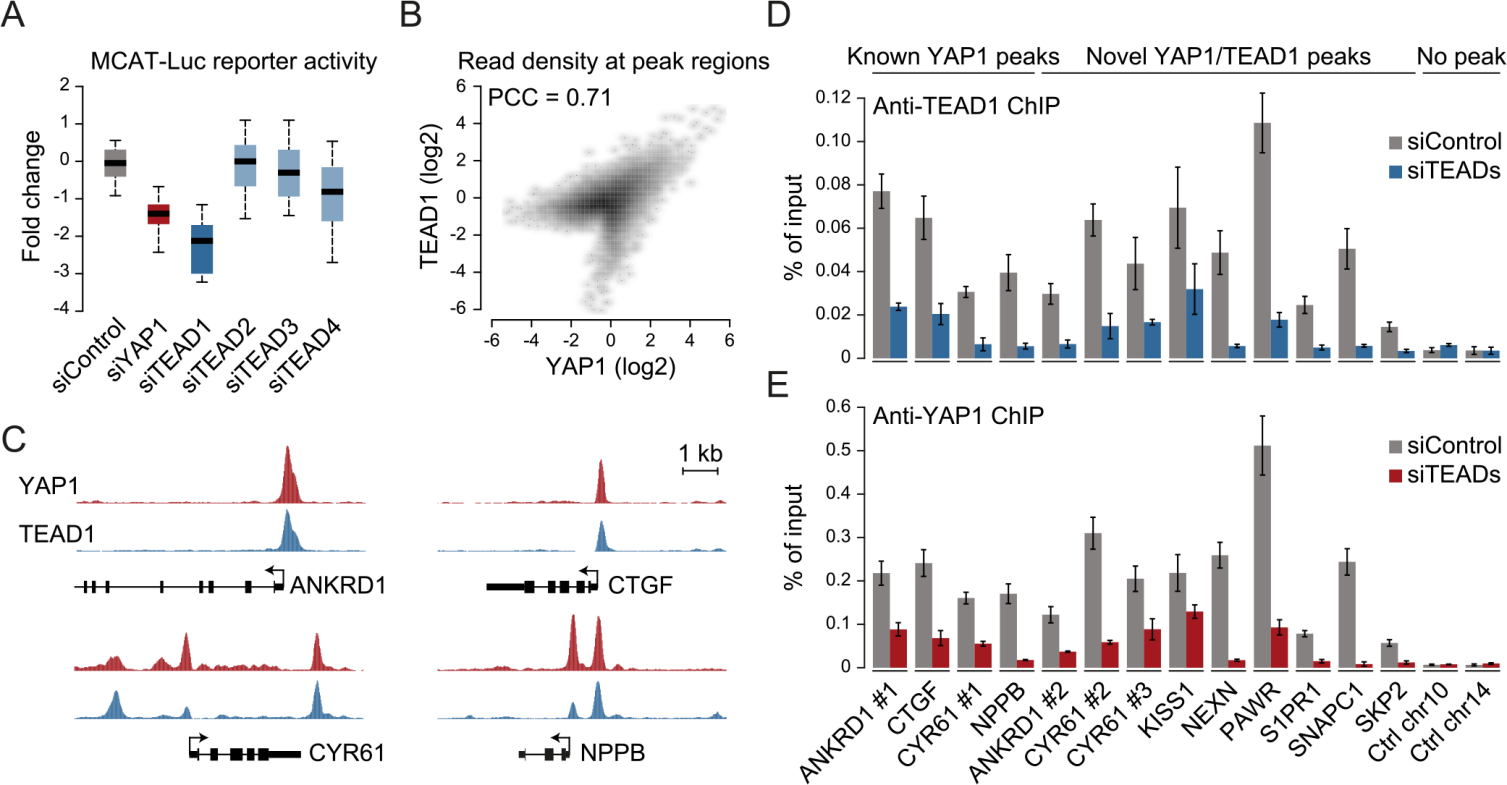
YAP1 peaks are co-occupied by TEAD1. (A) Expression changes of a YAP1/TEAD responsive luciferase reporter upon siRNA-mediated knockdown of YAP1 or TEADs normalized to a negative control siRNA in SF268 cells. (B) Correlation between TEAD1 and YAP1 SF268 ChIP-seq samples. (C) Genomic views of YAP1 and TEAD1 ChIP enrichment at gene promoters of known target genes. (D and E) Validation of (D) TEAD1 and (E) YAP1 binding to known and novel sites and control regions following siRNA depletion of TEADs as compared to control siRNA treated cells by ChIP-qPCR. Data are representative of at least three independent experiments. Error bars indicate the standard deviation of triplicate qPCR data.

The four different TEAD proteins display distinct expression patterns in cultured cell lines even though they have been suggested to be functionally redundant [54]. To establish which TEADs are essential for YAP1-mediated transcriptional activity in SF268 cells, we assessed the expression of the TEAD-dependent MCAT-luciferase reporter upon siRNA-mediated depletion of individual TEADs. This revealed that the depletion of TEAD1 had a potent effect on reporter gene activity, while knockdown of TEAD4 had only marginal effects (Fig 3A).

As TEAD1 appears to be the primary transcriptionally active TEAD family member in SF268 cells, we next mapped its genome-wide binding profile by ChIP-seq (S4 Fig). This led to the identification of 2,652 TEAD1 binding sites based on two independent, but highly reproducible biological replicates and matching input (S1 Table). We first noted a high similarity between TEAD1 and YAP1 ChIP samples, which is reflected in a high positive correlation (PCC = 0.71) (Fig 3B) and a remarkable overlap of 90% with YAP1 peaks regions (Fig 3C and S3 Fig). siRNA-mediated depletion of TEADs strongly reduced TEAD1 binding to all tested loci, thereby confirming the specific binding of TEAD1 to the identified peak regions (Fig 3D and S5 Fig). Reduction of TEADs also reduced YAP1 levels at all tested sites (Fig 3E). This further argues that YAP1 association with chromatin is mainly mediated via TEAD TFs and specifically by TEAD1 in the tested glioblastoma setting. Reciprocally, we observed that the majority of TEAD1 peaks overlap with YAP1 peaks arguing that all TEAD1 binding sites recruit YAP1.

### YAP1 and TEAD1 bind and activate distal enhancers

**Fig 4:**
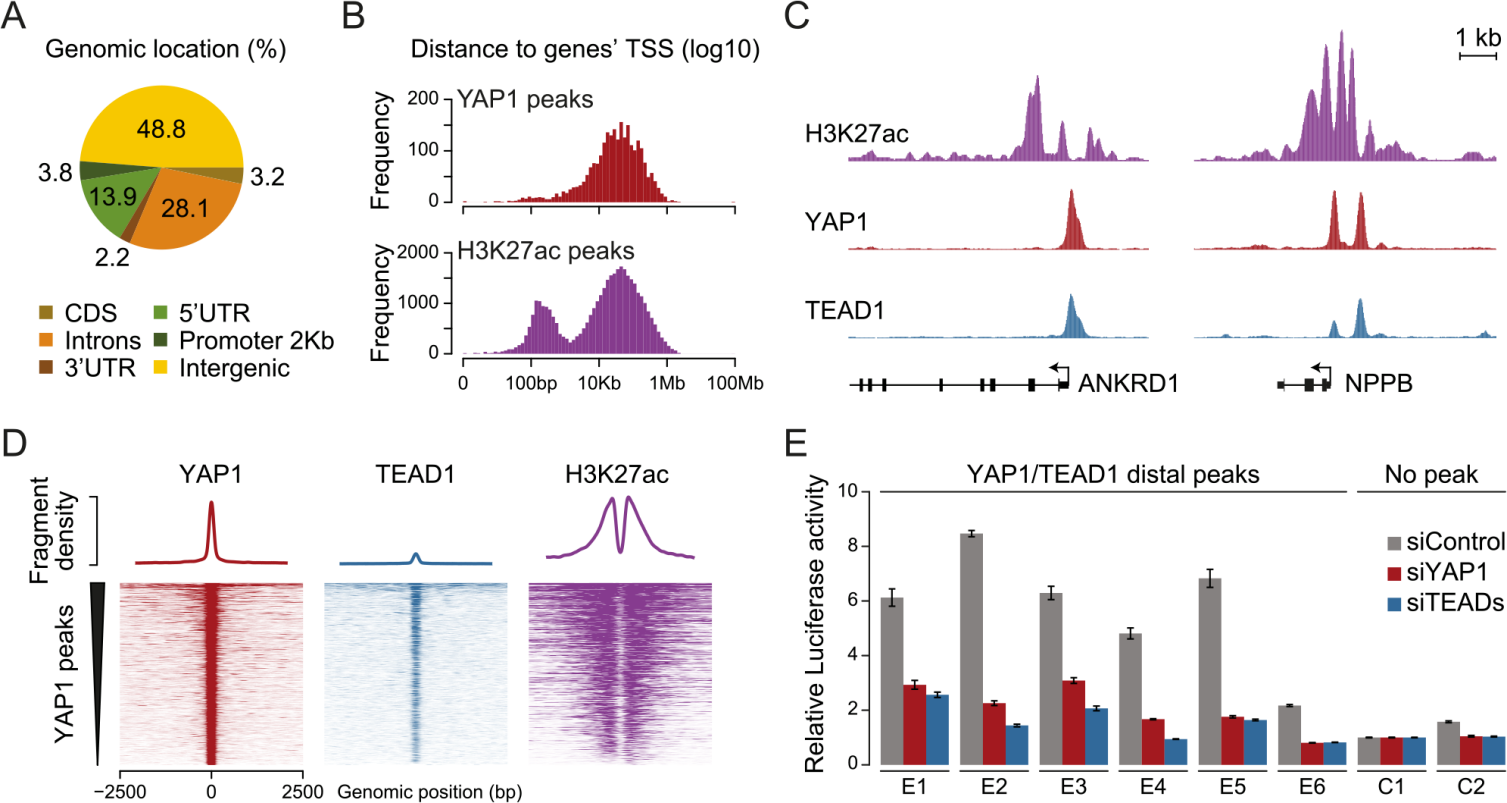
YAP1/TEAD1 associate with active enhancers. (A) Genomic distribution of YAP1/TEAD1 peaks. Promoter class defined as 2kb upstream of gene TSS. (B) Distance of YAP1/TEAD1 peaks and H3K27ac regions to closest gene TSS. (C) Genomic views of H3K27ac, YAP1 and TEAD1 ChIP enrichment at gene promoters of known target genes. (D) YAP1, TEAD1 and H3K27ac ChIP enrichment at all YAP1 peak regions centered on peak summit. (E) Luciferase reporter assay of six YAP1/TEAD1 distal enhancer binding sites containing single or double TEAD motifs in cells treated with YAP1 or TEADs siRNA compared to control siRNA. Data are representative of at least three independent experiments. Error bars indicate the standard deviation of triplicate qPCR data.

Previous studies focused primarily on the association of YAP1 with proximal promoters [22, 31, 33, 34, 36, 38, 55–57]. It is therefore not surprising that the majority of target genes described up to now contain a YAP1/TEAD1 peak in their promoter region. However, less than 4% of the YAP1/TEAD1 peaks identified in our study are actually located within 2Kb of a gene TSS, and only 15% are located in the 5’UTR of known genes (Fig 4A and S6 Fig). Thus, the majority of YAP1/TEAD1 binding sites reside distal to gene TSSs (Fig 4B, top) providing evidence that YAP1 acts at distal enhancers, which account for a large fraction of regulatory regions [58].

To evaluate whether these distal binding sites occur indeed within functional regions such as enhancers, we took advantage of the fact that acetylation at lysine 27 of histone H3 (H3K27ac) can serve as a signature mark of active enhancers [59, 60]. We performed ChIP-seq in SF268 cells using an H3K27ac-specific antibody in two independent biological replicates and matched input (S1 Table). This identified 38,331 H3K27ac positive regions both proximal and distal to gene TSSs (Fig 4B, bottom). Intersecting this dataset with YAP1 and TEAD1 reveals that 95% of the YAP1/TEAD1 peaks overlap with H3K27ac in particular on nucleosomes flanking YAP1/TEAD1 peaks (Fig 4C and 4D and S3 Fig). Thus, most of YAP1/TEAD1 binding appear to occur within active enhancers and likely represent functional binding events.

To test this hypothesis, we inserted several YAP1/TEAD1 occupied putative enhancer regions into reporter plasmids. In this experiment indeed five out of six tested elements were able to activate the transcription of a luciferase reporter. Notably, siRNA-mediated depletion of YAP1 or TEADs blunted their enhancer activity demonstrating their necessity for proper enhancer function (Fig 4E). These results provide experimental evidence that YAP1/TEAD1 bind primarily at active distal regulatory regions, contributing to enhancer activity.

### YAP1/TEAD1 regulate the H3K27ac enhancer chromatin mark

**Fig 5:**
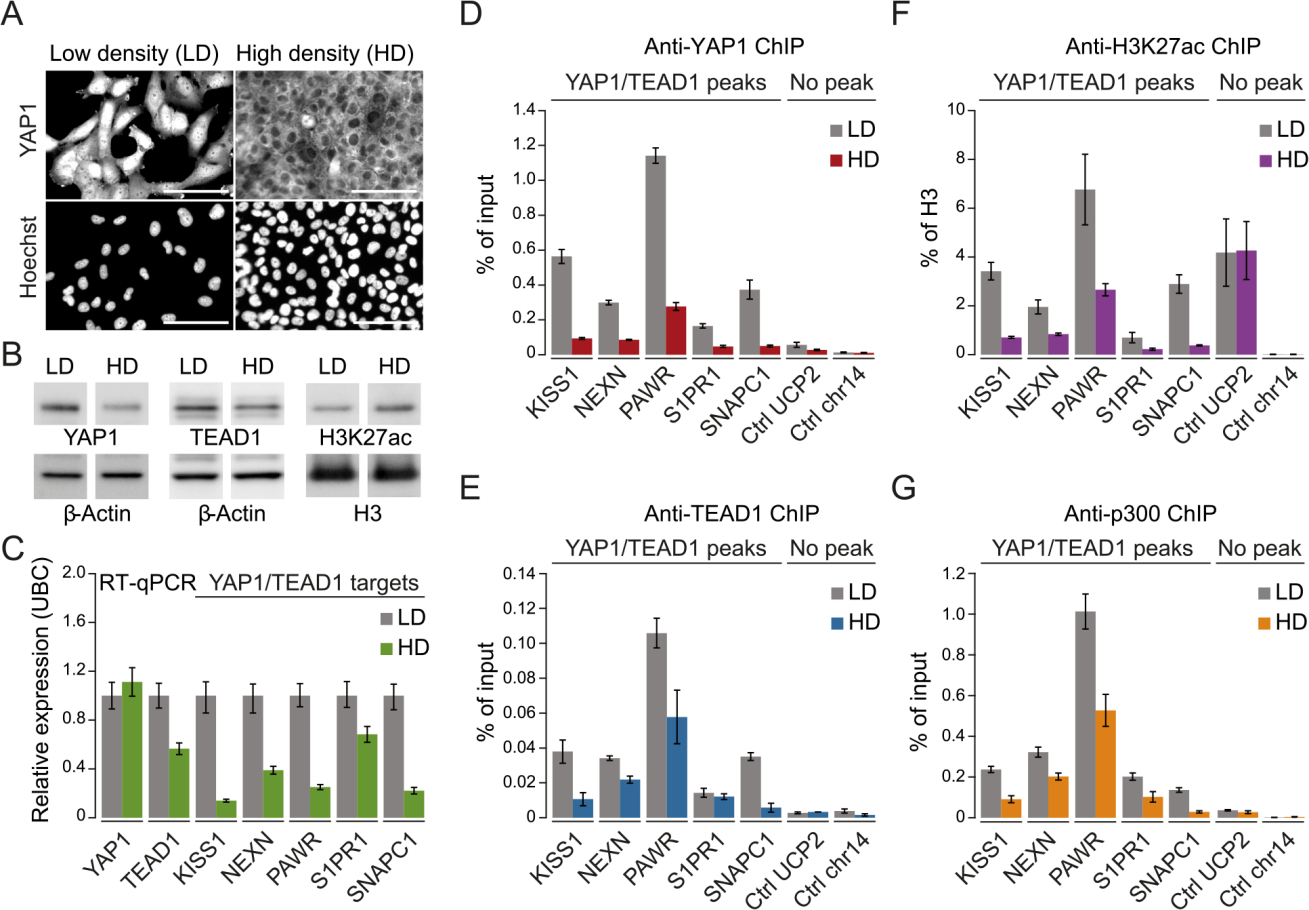
YAP1 mediates active enhancer chromatin and expression of target genes. (A) YAP1 immunofluorescence staining in SF268 cells grown at low (LD) or high density (HD). The corresponding DNA Hoechst 33342 staining is shown. Scale bar = 100μm. (B) Western blot analysis of YAP1, TEAD1 and H3K27ac from LD and HD SF268 cells. β-Actin and histone H3 served as loading controls. (C) mRNA expression of *YAP1*, *TEAD1*, *KISS1*, *NEXN*, *PAWR*, *S1PR1*, and *SNAPC1* from cells cultured at LD or HD (normalized to *Ubiquitin C* (*UBC*)). Data are representative of at least three independent experiments. Error bars indicate the standard deviation of triplicate qPCR data.(D, E, F and G) Analysis of (D) YAP1, (E) TEAD1, (F) H3K27ac, and (G) p300 occupancy at YAP1/TEAD1 peak regions from cells cultured at LD or HD by ChIP-qPCR. Data are representative of at least three independent experiments. Error bars indicate the standard deviation of triplicate qPCR data.

To gain further mechanistic insight into YAP1/TEAD1 transcriptional regulation, we assessed the impact of YAP1 inactivation on TEAD1 chromatin recruitment, target gene expression, and the H3K27ac enhancer chromatin mark. More specifically we took advantage of contact inhibition as a physiological impetus to control YAP1 activity [7]. Although SF268 cells overexpress *YAP1*, they are nevertheless fully responsive to contact inhibition. When cultivated at high density, YAP1 translocates to the cytoplasm and is degraded as reflected by decreased protein levels (Fig 5A and 5B). This results in reduced target gene expression (Fig 5C) and coincides with diminished YAP1 recruitment (Fig 5D). Interestingly, inactivation of YAP1 also leads to a reduction of *TEAD1* expression (Fig 5C), which resulted in reduced cellular TEAD1 protein levels (Fig 5B) and subsequently diminished chromatin occupancy (Fig 5E).

Importantly, YAP1 nuclear depletion also decreases H3K27ac at YAP1/TEAD1 peaks (Fig 5F). This observation appears highly specific since global H3K27ac levels were not affected and regions not bound by YAP1/TEAD1 showed no reduction (Fig 5B and 5F). To gain further mechanistic insight into how YAP1 affects H3K27ac we performed ChIP-qPCR for p300, the major histone acetyltransferase that has been linked to enhancers [61, 62]. This reveals that p300 indeed binds to YAP1 positive H3K27 acetylated sites **(** Fig 5G **)**. Next we asked if p300 recruitment to these sites is YAP1-dependent by testing p300 occupancy upon YAP1 inactivation under high cell density conditions. This revealed reduced p300 levels mirroring the reduction in H3K27 acetylation. To independently test the link between YAP1 activity and enhancer chromatin, we furthermore depleted YAP1 using siRNA, which similarly led to reduced chromatin binding (S7 Fig). In agreement with YAP1 inactivation by high cell density, siRNA-mediated depletion of YAP1 resulted in diminished H3K27ac levels, p300 occupancy, and reduced *TEAD1* expression and chromatin occupancy probably through disruption of a positive feedback loop (S3 and S7 Figs). Together, these data confirm the link between YAP1 chromatin binding and transcriptional activation of target genes and establish a requirement for YAP1 for proper chromatin structure at enhancers.

### YAP1 binds similar sites in YAP1-activated cancer cells

**Fig 6:**
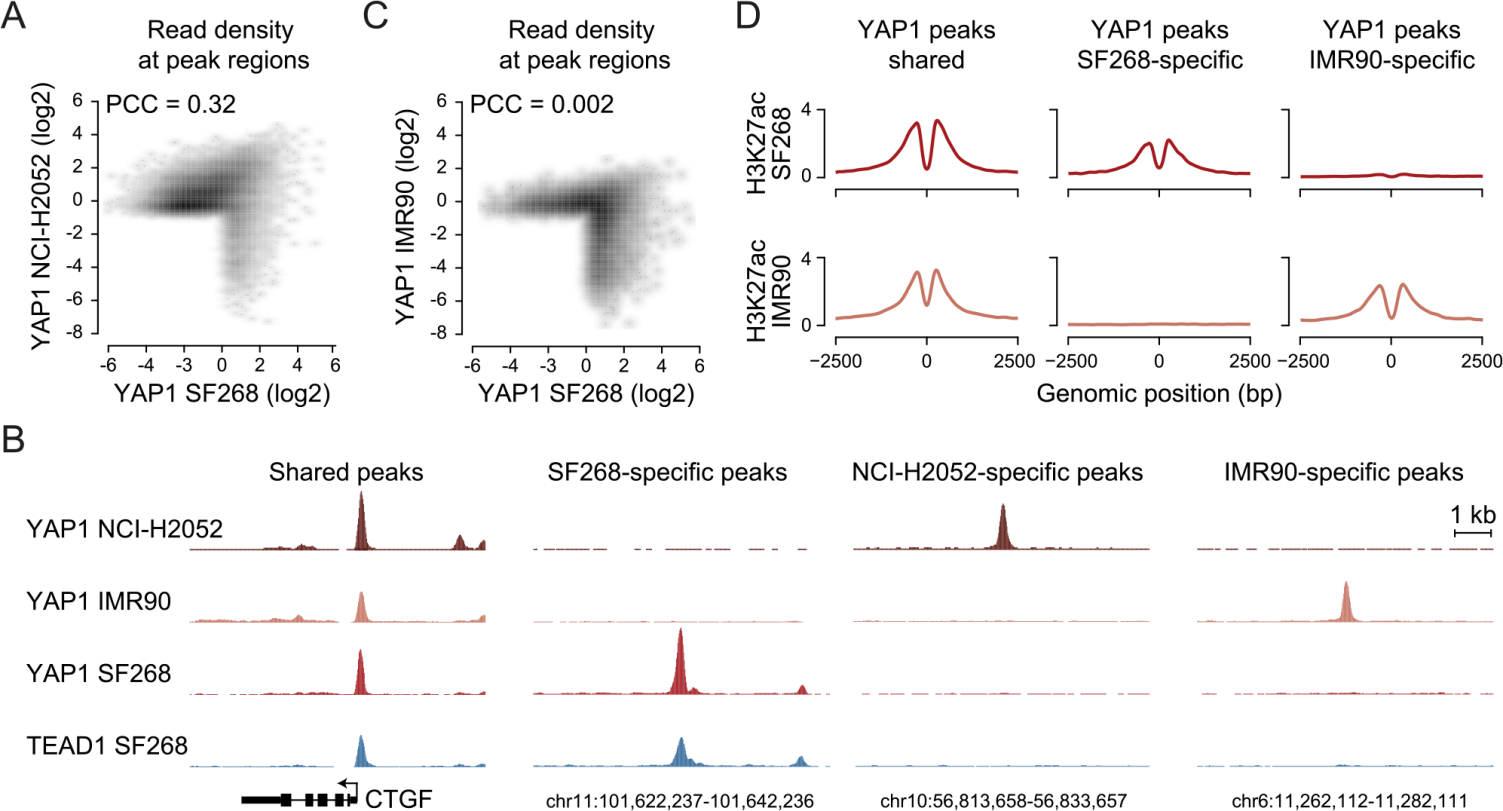
YAP1 binding sites largely overlap in cancer cell lines from distinct lineages. (A) Correlation between SF268 and NCI-H2052 YAP1 ChIP-seq samples. (B) Genomic views of YAP1 shared, SF268-, NCI-H2052 and IMR90-specific regions. (C) Correlation between SF268 and IMR90 YAP1 ChIP-seq samples. (D) H3K27ac ChIP enrichment at YAP1 peak regions (centered on peak summit) that are shared, SF268-specific or IMR90-specific.

Next, we asked if the observed YAP1 binding to distal enhancers is specific for cellular situations with extensive *YAP1* amplification such as in SF268 cells. Towards this goal we investigated YAP1 binding in NCI-H2052 malignant mesothelioma cells, a cell line of different lineage and with a different mechanism of YAP1 activation (*NF2* mutation, *LATS2* deletion) [63]. ChIP-seq analysis of YAP1 in two independent biological replicates and matching input identified 16,470 binding sites (S1 Table). This larger number of peak regions is due to many weak peaks that were not detected in SF268 cells. However, YAP1 binding is well conserved between SF268 and NCI-H2052 cells particularly at strong peaks. This is evident in a global positive correlation (PCC = 0.32) but also at the level of individual loci (Fig 6A and 6B). Indeed 82% of the YAP1 peaks identified in SF268 overlap with peaks in NCI-H2052 cells. As expected, we also identified 1,142 SF268- and 2,510 NCI-H2052-specific YAP1 binding sites using stringent thresholds but only 48% and 36%, respectively, were assigned to genes not targeted by shared peaks (S8 Fig) suggesting a common function for YAP1 in cancer cells. We also found that YAP1 binds mainly to distal regulatory regions in NCI-H2052 cells (S8 Fig) and that occupied sites were also enriched in TEAD single and double motifs as well as AP-1 motifs (S8 Fig). Similarly to SF268 cells, genetic knockdown of TEADs resulted in reduced YAP1 chromatin binding to NCI-H2052-specific and shared loci with SF268 cells, supporting that TEADs are the main mediators of YAP1 binding also in NCI-H2052 cells (S9 Fig).

Cell-type and context-dependent binding of TFs involves chromatin architecture and epigenetic modifications which are often altered during tumor development [64]. Thus, YAP1 binding in cancer cells might differ from non-transformed cells. To investigate whether YAP1 also binds primarily at TEAD-mediated enhancers in non-transformed cells, we investigated its binding profile in non-transformed lung fibroblast cells (IMR90) as primary cells for which many genomic datasets exist [65, 66]. ChIP-seq analysis of YAP1 in two independent biological replicates and matching input identified 1,111 binding sites (S1 Table). Notably, we found no significant global correlation (PCC = 0.002) of YAP1 binding profiles between SF268 and IMR90 (Fig 6B and 6C). Indeed only 42% of YAP1 peaks in SF268 overlapped peaks in IMR90. This difference in binding also holds true at the gene level (S8 Fig). Despite these differences binding nevertheless takes place predominantly at distal regulatory regions (S8 Fig). Furthermore, cell type-specific binding generates cell type-specific presence of the H3K27ac mark suggesting that those sites are functional (Fig 6D). Finally, YAP1 binding sites in IMR90 are also predominantly enriched in TEAD motifs. Importantly, depletion of TEADs using siRNAs resulted in reduced YAP1 occupancy at cell type-specific and shared loci confirming the general observations made in the cancer cell lines (S9 Fig). Different from the cancer cell lines the consensus motif for forkhead TFs (FOX) is significantly enriched as a secondary motif at IMR90-specific sites (S8D Fig). This might indicate that FOX factors act as cell type-specific contributors to YAP1/TEAD transcriptional regulation. This is compatible with a recent publication that shows a functional interaction of YAP1 and FoxO1 in cardiomyocytes [67]. This however remains challenging to test experimentally due to the fact that more than 20 FOX TFs are expressed in these particular cells that are all predicted to bind to this consensus motif. While it remains to be determined if FOX TFs contribute to cell type-specific TEAD binding our data clearly reveal that also YAP1 binding in non-transformed cells is mainly mediated by TEAD (S8 Fig).

Taken together, these findings indicate that YAP1 binding to enhancers, as well as the presence of double TEAD motifs with a 3bp spacer, are general features of YAP1-mediated transcription in YAP1-activated cancer as well as non-transformed cells even though targeted enhancers can largely differ.

### A novel set of YAP1/TEAD1 target genes

**Fig 7:**
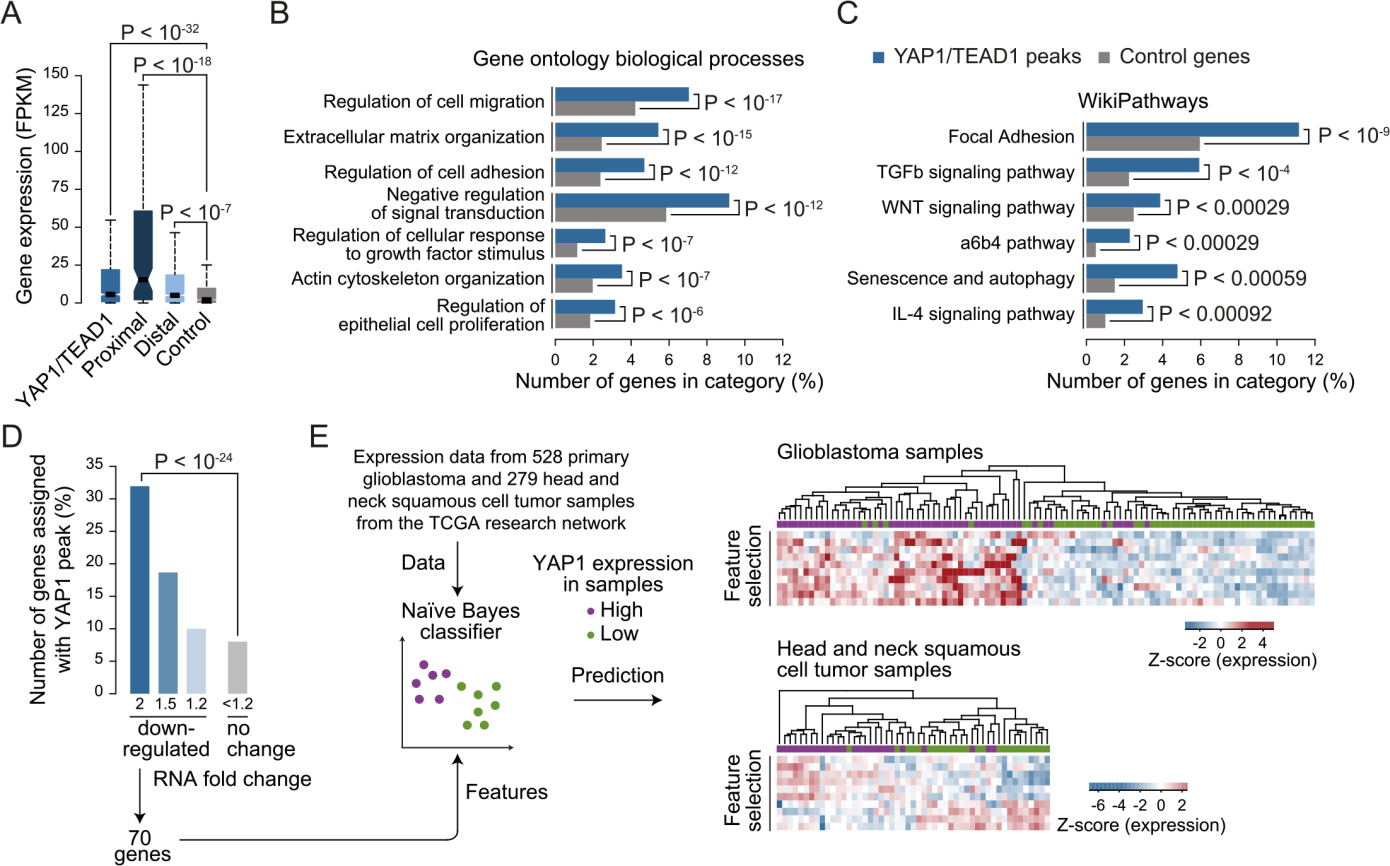
YAP1/TEAD1 target genes. (A) Gene expression of target genes from all, proximal (≤2Kb), distal (>2Kb) or random YAP1/TEAD1 peaks. Peaks were assigned to their closest gene TSS. (B and C) Gene enrichment analysis of YAP1/TEAD1 target genes for (B) gene ontology biological processes and (C) WikiPathways. (D) Number of genes at selected expression fold change also targeted by YAP1/TEAD1 peaks. (E) Prediction of *YAP1* expression (high: purple vs. low: green) in glioblastoma and head and neck squamous cell tumor samples using the gene features extracted from 70 genes 2-fold down-regulated in YAP1 siRNA knockdowns and targeted by a YAP1/TEAD1 peak.

The high occurrences of YAP1 binding sites that we identify at distal enhancers suggest that the number of direct YAP1 target genes is much larger than previously anticipated based on studies that focused on promoter regions. While it is undisputed that enhancers are highly relevant for gene activation it remains challenging to correctly assign their target genes due to the fact that enhancers can regulate genes over long distances [68]. Nevertheless, assigning enhancers to the gene in their nearest vicinity is a useful approximation that is correct in the majority of cases [62, 69]. Based on this observation we assigned each YAP1/TEAD1 peak to its nearest gene TSS, yielding 1,738 genes in SF268 cells (S2 Table). In agreement with the notion that YAP1 mainly functions as a transcriptional co-activator this gene set was expressed at a significantly higher level as compared to a random control set (Fig 7A) indicating that the peak-to-gene assignment is overall accurate. Distal YAP1/TEAD1 peaks (over 2Kb away from gene TSS) were assigned to 1580 genes, 52 of which also had a proximal peak. Importantly, the 1528 genes with only distal YAP1/TEAD1 peaks were also more highly expressed than a random control set (Fig 7A). To further, determine the accuracy of the peak to gene assignment we directly tested the expression of 19 randomly selected genes upon YAP1 or TEAD siRNA-mediated depletion (S10 Fig). Gene expression levels were affected in all tested cases arguing for a direct link between binding events and target gene expression.

Gene Ontology (GO) analysis of this gene set showed enrichment for previously reported YAP1 functions such as regulation of cell migration (hypergeometric *P* < 10^−17^), extracellular matrix organization (*P* < 10^−15^), actin cytoskeleton organization (*P* < 10^−07^), and regulation of epithelial cell proliferation (*P* < 10^−6^) (Fig 7B and S6 Table). In agreement with recent studies demonstrating a complex interaction network between the YAP1/Hippo and others signaling pathways such as WNT, BMP, TGF-β and PI3K-mTOR [70], our analysis reveals an enrichment of terms associated with signaling (Fig 7B and 7C and S6 Table). We noted that this set of genes contains a number of core components and downstream targets of diverse signaling pathways (S11 Fig). In addition, a number of YAP1/Hippo pathway components including *WWC1*, *LATS*, *NF2*, and *AMOT* are bound by YAP1/TEAD1. This suggests an extensive feedback mechanism in vertebrates and confirms previous reports in *Drosophila* [71, 72].

### Gene Signature Analysis predicts YAP1 levels in primary tumor samples

Next we set out to determine which genes are transcriptionally activated by YAP1 and performed RNA-seq profiling following YAP1 siRNA-mediated depletion in SF268 cells (Fig 7D and S12 Fig). This identified 219 and 360 genes that were down- or up-regulated by at least 2-fold, respectively, upon YAP1 knockdown compared to control siRNA-treated cells. Among the down-regulated genes, 70 (32%) contained a YAP1/TEAD1 peak assignment in SF268 cells (Fig 7D).

To evaluate the physiological relevance of these YAP1-activated target genes, we sought to predict YAP1 expression in tumor samples using expression data for 528 primary glioblastoma and 279 head and neck squamous cell tumor samples [73]. For each indication, we labeled the samples as *YAP1* “high” or “low” expression and divided the datasets into training and test datasets (2/3 and 1/3 of the samples respectively) over 1000 randomized iterations. Using a naïve Bayes classifier this allowed to predict *YAP1* expression level with high accuracy (an area under the receiver operating characteristics curve (AUC) = 0.83 for glioblastoma samples and AUC = 0.78 for head and neck squamous tumor samples) (S13 Fig). Feature selection allowed reducing the full YAP1 gene signature to ten genes without losing prediction performance. Hierarchical clustering of the samples shows consistent patterns of expression depending on *YAP1* “high” or “low” expression (Fig 7D). This result supports the use of the acquired gene signature to identify YAP1-activated cancers.

## Discussion

By providing a comprehensive account of YAP1 genomic binding and its impact on transcription this study establishes that transcriptional regulation of YAP1 target genes is predominantly mediated by TEAD binding to distal enhancers. In addition to demonstrating this mode of regulation we show that this activity entails the establishment of chromatin marks typical to enhancers linking YAP1 activity to H3K27ac. The identified distal regions enabled us to largely expand the set of YAP1 target genes, which we foresee to be a valuable source for functional studies and which we show to have predictive power to identify YAP1-activated cancers.

Due to lack of a DNA-binding domain, YAP1 requires TFs for genomic recruitment. TEAD family members are considered the main TFs for YAP1-mediated regulation of gene expression [22, 23, 25]. In support of a dominant function of TEADs in cancer cells, overexpression of an artificial TEAD2-VP16 construct in NIH3T3 cells was reported to mimic the effects of YAP1 overexpression at the transcriptional level and lead to cell transformation [23]. Furthermore, mutations in YAP1 that prevent binding to TEAD were shown to abolish YAP1-induced transcription and cell transformation in NIH3T3 and MCF10A cells [22].

Here, we comprehensively mapped YAP1 chromatin binding genome-wide in two different cancer cell lines and in non-transformed cells, enabling an unbiased assessment of the sequence features that direct YAP1-mediated regulation. Our genome-wide map of TEAD1 binding sites revealed that the vast majority of YAP1 binding sites were co-occupied by TEAD1 confirming the dominant role of TEAD factors in the control of YAP1 transcriptional activity. To the best of our knowledge this is the first study demonstrating a genome-wide co-occupancy of both factors in cancer cells. Our data extends the results from a previous ChIP-on-chip study that used a promoter specific microarray and demonstrated a comparable overlap of >80% for YAP1 and TEAD1 binding around start sites in MCF10A mammary epithelial cells [22].

Despite the major role of TEADs to mediate YAP1 co-activator activity, additional TFs are described to interact with YAP1 (reviewed in [12]). Our data, however, do not provide evidence for the importance of additional TFs in targeting YAP1 chromatin binding. We demonstrate that this finding is not just limited to a cellular situation where *YAP1* is amplified since we observe a similar predominant enrichment of TEAD motifs in YAP1 peak regions in a YAP1-activated NCI-H2052 cancer cell line as well as in non-transformed IMR90 cells.

TEAD1 also referred to as TEF-1, for transcription enhancer factor 1, was first cloned in HeLa cells as an activator of the simian virus 40 (SV40) “enhancer”, which is a short 72bp sequence element that is a component of the viral early promoter [54, 74, 75]. So far, however, binding and function at endogenous elements that by the current definition of an enhancer act distal to promoters had not been investigated.

Our genome-wide analysis of YAP1/TEAD1 binding indicates that the vast majority of endogenous sites, in cancer and non-cancer cells (SF268, NCI-H2052 and IMR90), are actually located within distal regulatory regions representing enhancer elements. This mimics the typical distribution of sequence-specific TFs and is in line with the concept that distal TF and co-activator binding are key determinants of enhancer activity and in turn cell-type specific gene expression patterns [39, 68, 76]. Recent efforts in mapping enhancers in different tissues revealed that the human genome contains up to several hundred thousand distal regulatory regions, most of which are cell-type specific [77]. Their misregulation can be highly disease relevant since mutations in these regions have extensively been associated with disease susceptibility [78].

Distal binding has not been reported for Yki (Yorki) the *Drosophila* homolog of YAP1 and it remains open if this reflects a functional difference or the organization of the smaller and gene denser fly genome [57, 79]. Similarly, enhancer binding of Yap1 has not been reported in mouse embryonic stem cells [55]. However, when reanalyzing an available list of Yap1 peak regions from Lin *et al*., we observed that a large fraction of these Yap1 binding sites are located in regions distal from promoters. In support of our data two recent reports demonstrated that YAP1/TEAD regulate transcription by binding to distal enhancers [67, 80]. Together this argues that YAP1 distal binding is a general feature of YAP1/TEAD-driven transcription activation also in non-transformed cells and is not an acquired feature of cancer cells.

Distinct chromatin modifications are associated with various aspects of gene expression. In particular H3K27ac was found to be an effective means to determine enhancer activity [59, 81]. Our data show that the vast majority of YAP1 binding sites overlap H3K27ac positive regions and that cell-type specific YAP1 sites match cell type specific H3K27ac regions. Interestingly we show that, in SF268 cells, YAP1 chromatin association is a prerequisite for the deposition of H3K27ac supporting the fact that YAP1 binding sites represent functional enhancers.

Interestingly, genome-wide binding analysis in *Drosophila* revealed a correlation between Yki chromatin binding and trimethylation of H3K4 (H3K4me3) [57]. Consistent with this finding, nuclear receptor coactivator 6 (Ncoa6), a subunit of the Trithorax-related H3K4 methyltransferase complex, has been identified as a Yki binding protein that is required for transcriptional regulation [79]. Importantly, besides the H3K4 methyltransferases, the mammalian Ncoa6 has been reported to enhance the activity of TFs by interacting with histone acetyltransferases CBP/p300 [82]. Whether this NCOA6 function possibly facilitates YAP1-dependent acetylation of H3K27 (H3K27ac) and which additional cofactors are recruited to trigger transcriptional activity warrants further investigations.

The determination of genes targeted by specific enhancers remains a challenge. We observed that only a minority of genes nearest to binding sites was transcriptionally affected by depletion of YAP1. Notably however, only 10–25% of TF binding events in higher eukaryotes contribute to the expression of the closest proximal gene in any given cell type. This is likely to be an underestimate given the nature of enhancers and the complexity of transcription regulatory networks [76]. Besides the uncertainty of assigning binding sites to target genes, enhancers function in a modular manner such that they contribute additively and redundantly to the expression of their target genes [76, 83]. Therefore YAP1/TEAD contribution to transcriptional activity might not be apparent at many target genes.

In addition to shedding light on basic principles of YAP1 transcriptional regulation, the identification of distal regulation as the primary means of YAP1 transcriptional control enabled us to identify an extended list of target genes based on both YAP1 chromatin binding and gene expression changes. This novel YAP1 signature from YAP1-amplified glioblastoma cells should have predictive potential for the identification of YAP1-dependent tumors.

## Materials and Methods

### Cell culture and transfections

SF268 cells (NCI DCTD tumor/cell line repository) were maintained in RPMI 1640 Medium, GlutaMAX supplement, 25 mM HEPES, 10% (v/v) fetal calf serum and 1 mM sodium pyruvate. NCI-H2052 cells (ATCC) were maintained in RPMI, 10% (v/v) fetal calf serum, 1% (v/v) non-essential amino acids, 1 mM sodium pyruvate. IMR90 cells (ATCC) were maintained in EMEM (Sigma M 4655) supplemented with 1% non-essential amino acids (NEAA) and 10% FBS. All media and supplements were from Life Technologies. To obtain low density (LD) or high density (HD) cultures, SF268 cells were plated at 10.000 cells/cm^2^ or 100.000 cells/cm^2^, respectively and harvested 48h or 96 hours after seeding. Transient transfections of SF268, IMR90 and NCI-H2052 cells with siRNA (final concentration: 25 nM) were performed using Lipofectamine RNAiMAX (Life Technologies). Transient transfection of SF268 cells with plasmid DNA was performed using Cell Avalanche Transfection Reagent (EZ Biosystems). Cells were harvested at 48 hours or 72 hours post-transfection.

### Antibodies

The following antibodies were used for western blot, immunoprecipitation and chromatin immunoprecipitation (ChIP): anti-YAP1 [EP1674Y] (ab52771), anti-KAT3B/p300 (ab14984), and anti-H3 (ab1791) from Abcam; anti-TEAD1 (610922) from BD Transduction Laboratories; anti-TEAD4 (ARP33426_P050) from Aviva Biosystems; anti-β-Actin (A2066) from Sigma-Aldrich; anti-H3K27ac (AM 39133) from Active Motif. YAP1 and TEAD1 antibodies for ChIP-seq were characterized in western blot, immunoprecipitation and ChIP-qPCR (S2 and S4 Figs).

### Chromatin immunoprecipitation (ChIP)

ChIP was essentially carried out as previously described [84], with slight modifications. Chromatin was sonicated for 14 minutes using a Covaris E210 (Settings: 5% duty cycle, intensity 4). 60μg of chromatin were incubated over night at 4°C with 5μg of the corresponding antibody and for 2 hours with preblocked (tRNA, BSA) Dynabeads protein G. DNA was purified using the Minielute PCR purification kit (Qiagen).

### ChIP-qPCR

Quantitative PCR was performed using Maxima SYBR Green / ROX qPCR Master Mix (Thermo Scientific) and the ViiA 7 Real-Time PCR System (Life Technologies) and 1/80th of the ChIP sample or 0.01% of input chromatin per PCR, respectively. Amplifications were performed in triplicate, and mean values were expressed as percentage input. Standard deviation was calculated from the triplicates, and error bars are indicated accordingly. Primers are listed in Table 1 and Table 2.

**Table 1:** Primers used for ChIP-qPCR.

Name	Forward primer	Reverse primer	Size (bp)	Genomic coordinates
ANKRD1 #1	GAGGGGAGGACAAGCTAACC	CGATGTGATCACCACCAAAG	83	chr10:92681001–92681083
CTGF	GCCAATGAGCTGAATGGAGT	CAATCCGGTGTGAGTTGATG	88	chr6:132272566–132272653
CYR61 #1	AGCAAACAGCTCACTGCCTT	ATGGTAGTTGGAGGGTCGTG	169	chr1:86045890–86046058
NPPB	TCTGGAATGCTGACCCTTCT	CTTGGGTGACTTCGTCATCA	96	chr1:11919755–11919850
ANKRD1 #2	ATGGCCTGCCACTTTGTTAC	TTTTCAGAACTGGGGTCTGG	96	chr10:92691054–92691149
CYR61 #2	CCCTTGGCTGTTATGAGGAA	CCTTGCATTCCTTTGCATTT	139	chr1:86049930–86050068
CYR61 #3	AGGAGTGAGAGAAGCAAGCG	TGCTTGTGAGCTTGTCATCC	118	chr1:86072824–86072941
KISS1	GCCGACCTGCTGTAGACAAT	CAAGGGCATCTACCTACCCA	142	chr1:204164957–204165098
NEXN	TTTAGGGCATGGCTCACTTC	AAGAGGGATTTTCATGGCCT	126	chr1:78567437–78567562
PAWR	CAGCATTCCTGTCATTCCCT	CAGGCTTCTTTTCTTGCACC	195	chr12:79941168–79941362
S1PR1	GTTCAGGATCAAGCTCCACC	GCTGAGAGCAGCCTGAGAAT	156	chr1:101666161–101666316
SNAPC1	TCTTCCAGCCTCTGCTCATT	CAGCTTGACTTTTCCCTTGG	100	chr14:62222431–62222530
SKP2	GCACAGAGGGAAACCAATGT	GTCCCTCATCCTGCATCACT	96	chr5:36156228–36156323
Ctrl chr10	ACCAACACTCTTCCCTCAGC	TTATTTTGGTTCAGGTGGTTGA	100	chr10:60902566–60902665
Ctrl chr14	GTGGGCCTTTGGAATATCCT	GACCTTGGCTGTGTTGTCCT	128	chr14:66894932–66895059

**Table 2:** Primers used for ChIP-qPCR (Figs 5 and S7 and S9).

Name	Forward primer	Reverse primer	Size (bp)	Genomic coordinates
KISS1	CTTTCCATCCTCCACACCCT	ACTAGGTGTGTCTGTGGCTC	145	chr1:204165829–204165973
NEXN	GGGCAGAAAGAGAAGGAGGA	GAACATCTGCTCGTGGGACA	120	chr1:78566826–78566945
PAWR	CAGCATTCCTGTCATTCCCT	CAGGCTTCTTTTCTTGCACC	195	chr12:79941168–79941362
S1PR1	AGCGGTAGTATCACATCTCTCT	TCTTCTCCTTTACTCCTGTTCTC	81	chr1:101666049–101666129
SNAPC1	TCTTCCAGCCTCTGCTCATT	CAGCTTGACTTTTCCCTTGG	100	chr14:62222431–62222530
IFIT1	TGACTGATCCTCCACACTGG	ACCATGACCACCATCCATCT	111	chr10:91157752–91157862
FAM150B	CAATGCCCATGTTTGTGTGT	GCAGTGGAGATGATCCAGGT	102	chr2:285749–285850
PLA2G16	TAAACAACCCCAAACCCTCA	TCACTCAAGGCTATGCAGGA	82	chr11:63376086–63376167
MFAP5	AGCAACTGCAAATTCCCATC	CCGCTGAAACAATGAGATGA	105	chr12:8815358–8815462
PPP2R5A	GTCCTAAGGGCAAACAGCAA	ATGAAACTGCCTGAGGATGC	143	chr1:212485630–212485772
DHTKD1	AAGCCCAAGACATCCTCCTT	AGGGCAGAAACTGTGCCTTA	95	chr10:12109374–12109468
NPC1	ACTGGGTTGGGAGGAGAAGT	TTTCAGCGAAGGGTAGTGCT	119	chr18:21166727–21166845
TCOF1	CTACCGACAGGGATTCCAAA	GCTCAACTTTGCCAGACACA	133	chr5:149814163–149814295
EPB41L2#1	AAGGGAGAAACGTTGGAGGT	TGGGTGGCATACACAGTTTG	138	chr6:131374839–131374976
EPB41L2#2	GGAATGACCTCAGTGTCTCAAA	TGACAGTCACCAGCAAAGGA	94	chr6:131175088–131175181
C1orf198	TGCCACATTCATGACATTCC	CAGGGTCTTTGCCTGGATAG	132	chr1:231005302–231005433
CCL2	GAAAGTGACTTGGCCTTTGC	AAGTGGGAGGCAGACAGCTA	109	chr17:29603882–29603990
RNF144A	CATACCAATGCTGGGTGAAA	ACCCAGTCTCCACACAAAGG	143	chr2:6684254–6684396
ITPR3	GGAAGGAGTCCAGTGGCTTA	ATGAGGGTCAGAAGGGAGGA	87	chr6:33685319–33685405
MAN1C1	TTCCCAATTCTGTCTCATGATCT	GTCTTGGTGGGAGGAAGTGA	120	chr1:26038242–26038361
Ctrl chr10	ACCAACACTCTTCCCTCAGC	TTATTTTGGTTCAGGTGGTTGA	100	chr10:60902566–60902665
Ctrl chr14	GTGGGCCTTTGGAATATCCT	GACCTTGGCTGTGTTGTCCT	128	chr14:66894932–66895059
Ctrl UCP2	GCGTTTACTCCTTCGTTCCC	AAGGCAAGAGGTGTGTGACT	145	chr11:73694084–73694228

### ChIP-seq

YAP1, TEAD1 and H3K27ac ChIPs from SF268 cells and YAP1 ChIPs from NCI-H2052 and IMR90 cells were subjected to high-throughput sequencing on a 356R Illumina HiSeq 2500 sequencer using standard NEB library preparation kits and protocols.

### ChIP-seq data processing

Additional ChIP-seq dataset for H3K27ac in IMR90 cells was obtained from the Gene Expression Omnibus under the accession number GSM469967. We mapped the ChIP-seq sequencing reads (single-end, 50bp) to the human reference genome (hg19 only chromosomes 1 to 22, X, Y and M) using bowtie [85] version 1.0.0 with parameters-v 3-m 1—best—strata. We extended the reads to 150bp (average estimated fragment length) and calculated for each genomic position the read density normalized to one million reads in the library to generate wiggle files. Genome screenshots were taken using the UCSC genome browser [86].

### Peak calling and overlap

We identified peaks in YAP1 and TEAD1 ChIP samples compared to the corresponding input samples using peakzilla [45] and in H3K27ac ChIP using MACS [87] version 1.4.2. The strategy used to define and overlap peak regions is described in [88] and S1 Table. We defined control peak regions by shuffling the peaks randomly within the same chromosome. We calculated the Pearson correlation coefficient (PCC) and plotted scatterplots between two samples using the mean fragment density of each peak region from all samples. Differentially bound regions were identified with the R package DESeq [89] using an adjusted p-value threshold of 10^−5^ and a 2-fold enrichment with enrichment in the reference sample below 100 normalized reads per kilobase.

### Motif enrichment analysis

We searched for motif de novo using MEME [46] within 31bp around peak summits and for occurrences of the known motifs from Jaspar [90], and [49] using MAST [91] (from the MEME suite programs version 4.1.1) with a P-value of 10^−3^ in an area of 151bp (average genomic fragment length) around each peak summit.

### Functional analyses

We assigned each peak to its closest gene transcriptional start site (TSS) using the reference transcriptome (GRCh37.71). For each gene ontology biological processes [92] and WikiPathways [93], we calculated the enrichment and associated hypergeometric P-values of genes in each class compared to all genes. We calculated the conservation rate of regions using the PhastCons 46 way placental mammals [94].

### RT-qPCR

RNA was isolated using RNeasy Mini Kit (Qiagen) and cDNA synthesis was performed using the High-Capacity RNA-to-cDNA Kit (Applied Biosystems). cDNA was subjected to quantitative PCR (qPCR) analysis in triplicates with gene-specific primers (see Table 3) using Maxima SYBR Green / ROX qPCR Master Mix (Thermo Scientific) and the ViiA 7 Real-Time PCR System (Life Technologies).

**Table 3:** Primers used for RT-qPCR.

Gene	Forward primer	Reverse primer	Size (bp)
CYR61	AAGAAACCCGGATTTGTGAG	GCTGCATTTCTTGCCCTTT	77
CTGF	CTCCTGCAGGCTAGAGAAGC	GATGCACTTTTTGCCCTTCTT	94
NPPB	GCTTTGGGAGGAAGATGGAC	GCAGCCAGGACTTCCTCTTA	88
YAP1	GCAAATTCTCCAAAATGTCAGG	CGGGAGAAGACACTGGATTT	94
UBC	AGGCAAAGATCCAAGATAAGGA	GGACCAAGTGCAGAGTGGAC	132
TEAD1	CTGAGTCGCAGTTACCACCA	AGCCTGGAGCCTTTTCAAG	92
TEAD2	ACATGATGAACAGCGTCCTG	CAGCAGTTCCTGGGTGTCTC	74
TEAD3	CATCGAGCAGAGCTTCCAG	CGTGCAATCAACTCATTTCG	111
TEAD4	GCCTTCCACAGTAGCATGG	AAAGCTCCTTGCCAAAACC	74
SNAPC1	GAATGAAAGTTTGAGTGGAACAGA	CCAGGCTCTTTGTTCAGTGTT	71
SKP2	CTGTCTCAAGGGGTGATTGC	TGTACACGAAAAGGGCTGAA	86
ANKRD1	TTTGGCAATTGTGGAGAAGTTA	AAACATCCAGGTTTCCTCCA	110
KISS1	GCCCACCCTCTGGACATT	CAGGTCCTAGAAGTGCCTTGA	111
DKK1	CAGGCGTGCAAATCTGTCT	AATGATTTTGATCAGAAGACACACATA	120
LGR5	CAGCGTCTTCACCTCCTACC	TCCAGGAAGCGGAGACTG	87
HAX1	AGAGTGATGCAAGAAGTGAATCC	GGGTCCATAGGCCATACATC	94
S1PR1	AACTTCGCCCTGCTTGAG	TCCAGGCTTTTTGTGTAGCTT	77
PAWR	CGTCCCCTACAAGCTCCTC	GATGCCAGGAGACGACCTC	83
NEXN	TGGAGAAACAAGAATTTGAACAAC	TGCTCAATCCAAAGGTTTCA	78
NTF3	CCCTTGTATCTCATGGAGGATT	TTTCCGCCGTGATGTTCT	66
BMP4	TCCACAGCACTGGTCTTGAG	GGGATGTTCTCCAGATGTTCTT	94
CALD1	CTGCTCCCAAACCTTCTGAC	GATTGCTTTTCCCAGAGGTTC	70
FAM171A	CCTGACCGCGTTTCTCAC	GTCATGCCTGGTGCTGTTT	106
ADRB2	CCATGTCCAGAACCTTAGCC	GATCTGCGGAGTCCATGC	63
GPR126	GGAACTACACGGTTTATGTCGTT	GGCTTCTCTTGACTTTAATCTTGTC	78
HAPLN1	AGTCTACTTCTTCTGGTGCTGATTT	TAGATGGGGGCCATTTTCT	114
KDR	GCTCAAGACAGGAAGACCAAG	GGTGCCACACGCTCTAGG	71
ZFP82	GCCCAGGGGGTAAAGAGAG	TCAGTCCTCCTTGGGGTTTA	75
CPA	TGACAGGGAGAAGGTGTTCC	GGCACCTGGATACCAGAAGT	106
PSG1	CGTTTCACCTTCACCTTACACC	GGAGTCTCAGGGTCACAGGTT	113
CCDC80	CAGGCGTGCAATTTTGGT	AATTGGGAACAGTTCTAACACTCC	93
PARVA	TCCTTCTTGGGGAAACTCG	CTCCTGCAGCTCGGACAC	72
VGLL4	ACTGCAACCTCTCGCACTG	GGAAATGCTCCTCCACCA	120
PLA2G16	TCTACGCAGCGAAATCGAG	AGGGCGAAAAATCTCAATCA	108
C1orf198	CAGAAGGTGGTGCGCTTC	ACTGAACTCCATCTGACTCTTTGTT	96
MFAP5	CCAGCCAAAGTAGGAACAGC	GGTCCCAAGAGCGACATATT	104
PPP2R5A	TGCTCAGCTAGCATATTGTGTTG	GCCAAAATTTCAGCAGTCCT	89
DHTKD1	TGTCGAAACTAATGCTGGAATC	TCCATATCGCTTCACTGTCG	77
NPC1	TTCGGCAGCTTCAGACACTA	TTCAGTAGGTTATAAAAACAGGATGG	88
TCOF1	GCAGGGAAGCAGGATGACT	TCATGGGATTCAAGAAGACTCC	111
EPB41L2	ACCATCAGGGAGGAACAGG	GTTTTTACCACTGGTGGCTTG	80
IFIT1	AGAACGGCTGCCTAATTTACAG	GCTCCAGACTATCCTTGACCTG	73
CCL2	CTGCTCATAGCAGCCACCTT	GCACTGAGATCTTCCTATTGGTG	106
FAM150B	GATGCGCCAGGCTTCTTAC	TGTACGGTCTGCTCACTGCT	74
RNF144A	TAAGCACAGCAGGACACCAG	TGGTCATCGCAGAACAGTCT	86
ITPR3	CCAACATGAACCTGGATCG	AGCATGCTGCTTGTCTTCC	73
MAN1C1	GAGGGCCGATGAGAGTCA	GCCAAGCAAACTGCATCAT	83

### RNA-seq

Total RNA of three biological replicates was extracted from SF268 cells 48h after transfection with two individual siRNAs targeting YAP1 and unspecific control siRNAs using the Total RNA purification kit from Norgen Biotek. RNAseq libraries were prepared using the Illumina TruSeq RNA Sample Prep kit v2 and sequenced using the Illumina HiSeq2500 platform (76-bp paired-end reads).

### RNA-seq data processing

Additional RNA-seq datasets for SF268 and LN229 cells were obtained from the Cancer Genomics Hub (https://browser.cghub.ucsc.edu). We mapped the RNA-seq sequencing reads (paired-end, 100bp) to the human reference transcriptome (GRCh37.71) using tophat [95] version 1.3.1 with parameter—no-novel-juncs. We calculated genes FPKMs (fragments per kilobase of transcript per million mapped reads) using cufflinks [96] version 2.0.2 with parameter-G using the reference transcriptome (GRCh37.71). Differentially expressed genes in YAP1 knockdown were identified with the R package DESeq [89]. Genes either down- or up-regulated were selected using an adjusted p-value threshold of 10^−5^ in all four pairwise comparisons of YAP1 and control siRNA treated samples and an at least 2-fold enrichment in one comparison and at least 1.2-fold in the other three.

### Luciferase reporter assays

GeneArt Strings DNA fragments encompassing approximately 200bp of six distal enhancers bound by YAP1/TEAD1 (see Table 4) and two negative regions carrying BglII restriction sites were cloned into pGL3 promoter vector (E1761, Promega) upstream of the luciferase gene with SV40 minimal promoter. For two regions mutations were introduced in either one or both motif sites of the double TEAD motif with 3bp spacer (see Table 5). One day prior transfection SF268 cells were plated on 384-well plates (1800 cells/well). Cells were co-transfected with 28.5 ng of the respective reporter constructs and 1.5 ng pRenilla.

**Table 4:** Luciferase reporters (Fig 4E).

Name	Closest gene	Distance to gene (bp)	Genomic coordinates	Size (bp)	TEAD motif
E1	S1PR1	36000	chr1:101666139–101666289	151	double
E2	PAWR	49133	chr12:79941090–79941289	200	double
E3	ANKRD1	9000	chr10:92690950–92691186	237	single
E4	NR2F2	434799	chr15:97311066–97311265	200	3 single
E5	JPH1	143000	chr8:75090094–75090360	267	4 single
E6	CCDC80	6000	chr3:112366054–112366253	200	double

**Table 5:** Luciferase reporters (Fig 2I).

Name	Closest gene	Distance to gene (bp)	Genomic coordinates	Sequence mutation (bold)	Size (bp)
No motif region #1			chr5:90656362–90656495		134
No motif region #2			chr1:20764655–20765577		152
Double motif region #1 wild type	S1PR1	36000	chr1:101666139–101666289	GGAATG-CAG-GGAATG	151
Double motif region #1 single mutant #1	S1PR1	36000	chr1:101666139–101666289	G**TG**A**GA**-CAG-GGAATG	151
Double motif region #1 single mutant #2	S1PR1	36000	chr1:101666139–101666289	GGAATG-CAG-**A**G**TGA**G	151
Double motif region #1 double mutant	S1PR1	36000	chr1:101666139–101666289	G**TG**A**GA**-CAG-**A**G**TGA**G	151
Double motif region #2 wild type	PAWR	49133	chr12:79941090–79941289	CATTCC-TGT-CATTCC	200
Double motif region #2 single mutant #1	PAWR	49133	chr12:79941090–79941289	**TC**T**CA**C-TGT-CATTCC	200
Double motif region #2 single mutant #2	PAWR	49133	chr12:79941090–79941289	CATTCC-TGT-C**TCA**C**T**	200
Double motif region #2 double mutant	PAWR	49133	chr12:79941090–79941289	**TC**T**CA**C-TGT-C**TCA**C**T**	200

For luciferase assays in YAP1 and TEADs-depleted cells, SF268 cells were transfected with the indicated siRNAs (see Table 6) at the day of seeding (1800 cells/well) in 384-well plates. The next day, the medium was changed and cells were transfected with DNA (pGL3 reporter constructs and pRenilla). Firefly and Renilla luminescence signals were measured at 24 hours after DNA transfection using Dual-Glo luciferase assay system (Promega). Firefly luminescence signals were normalized according to their corresponding Renilla signals resulting in relative luciferase activity. Each sample was transfected in triplicate, and each experiment was repeated independently at least three times.

**Table 6:** siRNAs.

Name	Target	Official name	Catalogue number	Vendor
siControl #1		ON-target plus siNontargeting#2	D-001810-02	Thermo Scientific
siControl #2		siAllStars negative control	SI03650318	Qiagen
siYAP1 #1	YAP1	ON-TARGETplus YAP1 #8	J-012200-08	Thermo Scientific
siYAP1 #2	YAP1	ON-TARGETplus YAP1 #7	J-012200-07	Thermo Scientific
siYAP1 #3	YAP1	YAP1_5 FlexiTube siRNA	SI02662954	Qiagen
siTEAD1 #1	TEAD1	ON-TARGETplus TEAD1 siRNA	J-012603-08	Thermo Scientific
siTEAD1 #2	TEAD1	TEAD1_5 FlexiTube siRNA	SI04181261	Qiagen
siTEAD2 #1	TEAD2	ON-TARGETplus TEAD2 siRNA	J-012611-09	Thermo Scientific
siTEAD2 #2	TEAD2	TEAD2_6 FlexiTube siRNA	SI04190249	Qiagen
siTEAD3 #1	TEAD3	ON-TARGETplus TEAD3 siRNA	J-012604-05	Thermo Scientific
siTEAD3 #2	TEAD3	TEAD3_7 FlexiTube siRNA	SI04329010	Qiagen
siTEAD4 #1	TEAD4	ON-TARGETplus TEAD4 siRNA	J-019570-08	Thermo Scientific
siTEAD4 #2	TEAD4	TEAD4_7 FlexiTube siRNA	SI04301346	Qiagen

SF268 cells stably expressing the MCAT-Luc YAP1/TEAD responsive reporter [44] were transfected with siRNAs targeting YAP1, TEAD1, TEAD2, TEAD3, and TEAD4 with 8 siRNAs per gene (see Table 7). At 72 hours after transfection medium was aspirated and cells were incubated with fresh medium containing 1.4μM resazurin (SIGMA; MO, USA) for 2 hours before measuring fluorescence (Ex: 540 nm, Em: 590 nm) as a read-out for cell viability. Subsequently the cells were lysed in fresh medium containing 1:10 (v/v) Steady-Glo luciferase assay reagent (Promega; WI, USA). Luciferase measurements were taken according to the manufacturer's protocol. Fold change in MCAT-Luc reporter activity was calculated by normalizing luminescence signal to resazurin and to negative control siRNA. Each experiment was carried out in triplicate.

**Table 7:** siRNAs (Fig 3A).

Target	Official name	Catalogue number	Vendor
TEAD1	ON-TARGETplus TEAD1 siRNA	J-012603-06	Dharmacon
TEAD1	ON-TARGETplus TEAD1 siRNA	J-012603-08	Dharmacon
TEAD1	ON-TARGETplus TEAD1 siRNA	J-012603-05	Dharmacon
TEAD1	ON-TARGETplus TEAD1 siRNA	J-012603-07	Dharmacon
TEAD1	TEAD1_8 FlexiTube siRNA	SI04279618	Qiagen
TEAD1	TEAD1_5 FlexiTube siRNA	SI04181261	Qiagen
TEAD1	TEAD1_6 FlexiTube siRNA	SI04237205	Qiagen
TEAD1	TEAD1_7 FlexiTube siRNA	SI04267200	Qiagen
TEAD2	ON-TARGETplus TEAD2 siRNA	J-012611-10	Dharmacon
TEAD2	ON-TARGETplus TEAD2 siRNA	J-012611-09	Dharmacon
TEAD2	ON-TARGETplus TEAD2 siRNA	J-012611-12	Dharmacon
TEAD2	ON-TARGETplus TEAD2 siRNA	J-012611-11	Dharmacon
TEAD2	TEAD2_8 FlexiTube siRNA	SI04360993	Qiagen
TEAD2	TEAD2_7 FlexiTube siRNA	SI04211704	Qiagen
TEAD2	TEAD2_5 FlexiTube siRNA	SI04178188	Qiagen
TEAD2	TEAD2_6 FlexiTube siRNA	SI04190249	Qiagen
TEAD3	ON-TARGETplus TEAD3 siRNA	J-012604-06	Dharmacon
TEAD3	ON-TARGETplus TEAD3 siRNA	J-012604-05	Dharmacon
TEAD3	ON-TARGETplus TEAD3 siRNA	J-012604-08	Dharmacon
TEAD3	ON-TARGETplus TEAD3 siRNA	J-012604-07	Dharmacon
TEAD3	TEAD3_8 FlexiTube siRNA	SI04375777	Qiagen
TEAD3	TEAD3_6 FlexiTube siRNA	SI04259570	Qiagen
TEAD3	TEAD3_5 FlexiTube siRNA	SI04207287	Qiagen
TEAD3	TEAD3_7 FlexiTube siRNA	SI04329010	Qiagen
TEAD4	ON-TARGETplus TEAD4 siRNA	J-019570-09	Dharmacon
TEAD4	ON-TARGETplus TEAD4 siRNA	J-019570-10	Dharmacon
TEAD4	ON-TARGETplus TEAD4 siRNA	J-019570-11	Dharmacon
TEAD4	ON-TARGETplus TEAD4 siRNA	J-019570-08	Dharmacon
TEAD4	TEAD4_5 FlexiTube siRNA	SI04131127	Qiagen
TEAD4	TEAD4_6 FlexiTube siRNA	SI04136069	Qiagen
TEAD4	TEAD4_8 FlexiTube siRNA	SI04360020	Qiagen
TEAD4	TEAD4_7 FlexiTube siRNA	SI04301346	Qiagen
YAP1	ON-TARGETplus YAP1	J-012200-06	Dharmacon
YAP1	ON-TARGETplus YAP1	J-012200-07	Dharmacon
YAP1	ON-TARGETplus YAP1	J-012200-08	Dharmacon
YAP1	ON-TARGETplus YAP1	J-012200-05	Dharmacon
YAP1	YAP1_1 FlexiTube siRNA	SI00084546	Qiagen
YAP1	YAP1_5 FlexiTube siRNA	SI02662954	Qiagen
YAP1	YAP1_4 FlexiTube siRNA	SI00084567	Qiagen
YAP1	YAP1_3 FlexiTube siRNA	SI00084560	Qiagen

### Protein isolation and western blot analysis

SF268 cells were lysed in FT lysis buffer (20 mM Tris / HCl at pH 7.8, 600 mM NaCl, 20% glycerol, proteinase inhibitor), and proteins including histones were extracted by repeated freeze-thaw cycles followed by Benzonase (Novagen) treatment. Lysates were separated using Novex NuPAGE SDS-PAGE gel system transferred to Immobilon-P membranes (Millipore) and subjected to immunoblotting.

### Immunofluorescence

Cells were fixed with 4% PFA (Paraformaldehyde 20% solution, EM grade #15713-S) for 15 minutes at room temperature. Subsequently cells were washed 1x PBS and permeabilized in PBS / 0.1% Triton X-100 at room temperature for 10 minutes. Cells were rinsed in PBS and incubated with anti-YAP1 diluted 1:300 in PBS / 1.5% BSA over-night at 4°C. Cells were washed with PBS and incubated with the secondary antibody, anti-rabbit Alexa647 (1:1000, Life Technologies) and Hoechst (1:10.000) for DNA staining for 2 hours at room temperature. After washing with PBS, staining was analyzed by fluorescence microscopy (Operetta, Perkin Elmer), 20x objective.

### Expression analysis in tumor samples

We used as gene signature 70 genes that were 2-fold down-regulated in YAP1 depleted SF268 cells and had a YAP1/TEAD1 binding peak in their vicinity. Expression data were collected from cBioPortal [73, 97] for 528 primary glioblastoma and 279 Head and neck squamous cell tumor samples generated by the TCGA Research Network. We used either all or the top and bottom 10% of samples according to the ranksum for *YAP1* expression and copy number. Over 1000 iterations we randomly divided the datasets into training and test subsets (2/3 and 1/3 respectively) and used a naïve Bayes predictor from the Bioconductor package e1071 to predict the *YAP1* expression level (“high” or “low”). Prediction accuracy was measured using recall statistics and receiver-operating-characteristic (ROC) curves and performance statistics were generated using the ROCR package [98].

### Accession numbers

Raw and processed ChIP-seq data are deposited in the Gene Expression Omnibus (GEO) under the accession number GSE61852. The raw RNA-seq reads are available in the NCBI Short Read Archive under the accession number SRP056665.

## Supporting Information

S1 Fig YAP1 protein levels in glioblastoma cell lines with (SF268) or without (LN229) *YAP1* amplification. (EPS)

S2 Fig YAP1 antibody validation. (A) Western blot analysis of YAP1 protein levels in control siRNA and YAP1 siRNA treated cells. β-Actin served as a loading control. (B) Immunoprecipitation (IP) efficiency of the YAP1 antibody determined by Immunoprecipitation (IP) followed by WB (IP-WB). S: supernatant after IP, E: eluat. (C) Validation of YAP1 binding to *CYR61* and *ANKRD1* promoters and a negative control region by ChIP-qPCR. Data are representative of at least three independent experiments. Data are shown as percent of input and error bars indicate the standard deviation of triplicate qPCR data.
(EPS)

S3 Fig Genomic views of YAP1, TEAD1, and H3K27ac peaks at *YAP1* and *TEAD1* genomic regions and at known YAP1/TEAD1 target genes. (EPS)

S4 Fig TEAD1 antibody validation. (A) Western blot analysis of TEAD1 protein level in cells treated with control siRNA and TEADs siRNA. β-Actin was used as a loading control. (B) IP efficiency of the TEAD1 antibody tested by IP-WB. S: supernatant after IP, E: eluat. (C) Validation of TEAD1 binding to *CYR61* and *ANKRD1* promoters and a negative control region by ChIP-qPCR. Data are representative of at least three independent experiments. Data are shown as percent of input and error bars indicate the standard deviation of triplicate qPCR data.
(EPS)

S5 Fig TEADs knockdown efficiency. (A) Western blot analysis of TEAD1 and TEAD4 protein levels in SF268 cells 48h after siRNA transfection. TEAD2 and TEAD3 protein levels were not detectable. (B) Expression analysis of *TEAD1*, *TEAD2*, *TEAD3* and *TEAD4* determined 48h after siRNA transfection by RT-qPCR (normalized to *UBC*). Data are representative of at least three independent experiments. Error bars indicate the standard deviation of triplicate qPCR data.
(EPS)

S6 Fig Distribution of genomic features in the genome. Promoter class defined as 2kb upstream of gene TSS.
(EPS)

S7 Fig siRNA-mediated depletion of YAP1 affects target gene expression and TEAD1 and H3K27ac chromatin occupancy. (A) Knockdown efficiency of *YAP1* on mRNA level and expression of *TEAD1*, *KISS1*, *NEXN*, *PAWR*, *S1PR1*, and *SNAPC1* upon siRNA-mediated YAP1-depletion determined by RT-qPCR (72h; normalized to *UBC)*. Data are representative of at least three independent experiments. Error bars indicate the standard deviation of triplicate qPCR data. (B, C and D) Western blot analysis of (B) YAP1, (C) TEAD1, and (D) H3K27ac upon siRNA-mediated YAP1-depletion (72h). β-Actin and histone H3 served as loading controls. (E, F, G and H) Analysis of (E) YAP1, (F) TEAD1,(G) H3K27ac, and (H) p300 occupancy following siRNA-mediated YAP1 depletion (72h) at YAP1/TEAD1 peak regions and control regions by ChIP-qPCR. Data are representative of at least three independent experiments. Data are shown as percent of input and error bars indicate the standard deviation of triplicate qPCR data.
(EPS)

S8 Fig YAP1-binding sites in IMR90 and NCI-H2052 cells. (A) Overlap of genes assigned from shared or of cell type specific peaks. (B) Distance of YAP1 peaks to closest gene TSS. (C) Number of peaks with motif. (D) number of peaks with forkhead box factor motif. (E) TEAD1-4 expression level in SF268, NCI-H2052 and IMR90 cells measured by RNA-seq.
(EPS)

S9 Fig YAP1 chromatin binding is mediated by TEAD in IMR90 and NCI-H2052 cells. (A) TEAD dependent expression of potential target genes assessed by RT-qPCR upon siRNA-mediated depletion of TEADs (72h; normalized to *UBC)* in NCI-H2052 cells. (B and C) Validation of (B) YAP1 and (C) TEAD1 binding to shared and cell type-specific sites and a negative control region following siRNA depletion of TEADs in NCI-H2052 cells as compared to control siRNA treated cells by ChIP-qPCR. (D) TEAD dependent expression of potential target genes assessed by RT-qPCR upon siRNA-mediated depletion of TEADs (72h; normalized to *UBC)* in IMR90 cells. (E and F) Validation of (E) YAP1 and (F) TEAD1 binding to shared and cell type-specific sites and a negative control region following siRNA depletion of TEADs in IMR90 cells as compared to control siRNA treated cells by ChIP-qPCR. Data are representative of at least two independent experiments. Error bars indicate the standard deviation of triplicate qPCR data.
(EPS)

S10 Fig Validation of YAP1/TEAD target genes. (A) Knockdown efficiency of *YAP1*, *TEAD1*, *TEAD2*, *TEAD3*, and *TEAD4* on mRNA level upon siRNA-mediated YAP1-depletion determined by RT-qPCR (72h; normalized to *UBC)*. Data are representative of at least three independent experiments. Error bars indicate the standard deviation of triplicate qPCR data. (B) YAP1 and TEAD dependent expression of potential target genes assessed by RT-qPCR upon siRNA-mediated depletion of YAP1 and TEADs (72h; normalized to *UBC)*. Data are representative of at least three independent experiments. Error bars indicate the standard deviation of triplicate qPCR data.
(EPS)

S11 Fig YAP1 targets key components of various signaling pathways. (EPS)

S12 Fig RNA-seq expression analysis. (A) Volcano plots of gene expression fold change versus adjusted p-value in all four YAP1 and control siRNA comparisons. (B) Reproducibility of fold change across YAP1 and control siRNA expression samples. (C) Knockdown efficiencies of YAP1 48h after siRNA transfection determined by RT-qPCR (normalized to *UBC*). Error bars indicate the standard deviation of triplicate qPCR data.
(EPS)

S13 Fig Receiver-operating-characteristic (ROC) curves. Receiver-operating-characteristic (ROC) curves for glioblastoma and head and neck squamous cell tumor samples.
(EPS)

S1 Table Peak calling strategy for ChIP-seq datasets. (PDF)

S2 Table YAP1/TEAD1 binding sites in SF268, NCI-H2052 and IMR90 cells. (XLSX)

S3 Table TEAD motif preferences. (PDF)

S4 Table Known motifs enriched in YAP1/TEAD1 peak regions. (PDF)

S5 Table Enriched motif logos. (PDF)

S6 Table Gene enrichment analyses in SF268 cells. (XLSX)

S7 Table YAP1-target genes in SF268 cells. (PDF)
