# Supplementary material for: YAP1 Exerts Its Transcriptional Control via TEAD-Mediated Activation of Enhancers
Source: PLoS Genet. 2015 Aug 21;11(8):e1005465. doi: 10.1371/journal.pgen.1005465 (PMC4546604; doi:10.1371/journal.pgen.1005465)
Supplement: S1 Table — (PDF) [file pgen.1005465.s014.pdf]

ChIP sample	Input sample	Peak calling software	Confident regions threshold	Enriched regions threshold	Peaks (replicates' agreement)
YAP1_ SF268_1	Input _ SF268_1	Peakzilla	2680	7659	2498
YAP1_ SF268_2			984	3397	
TEAD1_ SF268_1	Input _ SF268_2	Peakzilla	2683	8521	2652
TEAD1_ SF268_2			2249	7102	
H3K27ac_ SF268_1	Input _ SF268_2	MACS	35517	45205	38331
H3K27ac_ SF268_2			37041	46381	
YAP1_ NCI-H2052_1	Input_ NCI-H2052_1	Peakzilla	16388	33114	16470
YAP1_ NCI-H2052_2			13113	29142	
YAP1_ IMR90_1	Input_ IMR90_1	Peakzilla	3553	409	1111
YAP1_ IMR90_2			6884	1116	

S1 Table. Peak calling strategy for ChIP-seq datasets.

For YAP1 and TEAD1 datasets, confident regions were selected using peakzilla with score ≥ 2 and fold enrichment over input ≥ 4 and enriched regions with score ≥ 1 and fold enrichment over input ≥ 4. For H3K27ac datasets, confident regions were selected using MACS with P-value ≤ 10^{-10} and enriched regions with P-value ≤ 10^{-30}. We defined peaks as shared between samples if confident peaks called in one sample are overlapping an enriched region in another sample and reciprocally. This ensures that a peak that is enriched in both replicates but which missed the stringent threshold for peak calling in one of them is not wrongly discarded.
