# Supplementary material for: YAP1 Exerts Its Transcriptional Control via TEAD-Mediated Activation of Enhancers
Source: PLoS Genet. 2015 Aug 21;11(8):e1005465. doi: 10.1371/journal.pgen.1005465 (PMC4546604; doi:10.1371/journal.pgen.1005465)
Supplement: S3 Table — (PDF) [file pgen.1005465.s016.pdf]

6-mer	Total number of peaks	Number of peak regions with motif	Number of control peak regions with motif	Percent of peak regions with motif	Enrichment over control	P-value
CATTCC preferences	2498	2152	943	86.15	2.28	4.36×10^{-289}
CATTCC	2498	1333	206	53.36	6.47	1.18×10^{-284}
ACATTC	2498	1011	204	40.47	4.96	1.21×10^{-167}
ATTCCA	2498	1041	263	41.67	3.96	1.27×10^{-146}
ATTCCT	2498	1042	289	41.71	3.60	6.39×10^{-135}
GCATTC	2498	823	164	32.95	5.02	6.97×10^{-131}
AATTCC	2498	707	214	28.30	3.30	4.31×10^{-76}
CATTCT	2498	727	317	29.10	2.29	1.10×10^{-47}
ATTCCC	2498	449	179	17.97	2.51	4.30×10^{-32}
GATTCC	2498	331	118	13.25	2.80	2.05×10^{-27}
ATTCCG	2498	135	19	5.40	7.10	1.59×10^{-24}
TCATTC	2498	460	272	18.41	1.69	1.51×10^{-14}
CCATTC	2498	314	175	12.57	1.79	9.93×10^{-12}
CATACC	2498	255	131	10.21	1.95	1.21×10^{-11}
CATCCC	2498	302	170	12.09	1.78	4.83×10^{-11}
CCTTCC	2498	392	242	15.69	1.62	5.80×10^{-11}
CATTCA	2498	416	264	16.65	1.58	1.18×10^{-10}
CATTTC	2498	453	318	18.13	1.42	4.71×10^{-8}
CGTTCC	2498	71	24	2.84	2.96	1.86×10^{-7}
CATTGC	2498	280	184	11.21	1.52	1.05×10^{-6}
CATGCC	2498	389	284	15.57	1.37	5.41×10^{-6}
CAGTCC	2498	206	139	8.25	1.48	7.09×10^{-5}
CTTTCC	2498	353	276	14.13	1.28	4.34×10^{-4}
CATTCG	2498	53	26	2.12	2.04	6.72×10^{-4}
CATTAC	2498	150	144	6.00	1.04	0.34
CAATCC	2498	131	133	5.24	0.98	0.52
CACTCC	2498	265	275	10.61	0.96	0.66
TATTCC	2498	165	228	6.60	0.72	0.99

S3 Table. TEAD motif preferences.

The CATTCC motif preferences (first line) were calculated with the occurrences of the CATTCC 6-mers allowing for one mismatch to CATTCC using only the 6-mers that were present in at least 20% of the peaks and two fold enriched over control peaks (seven 6-mers in the second section).
