# Supplementary material for: YAP1 Exerts Its Transcriptional Control via TEAD-Mediated Activation of Enhancers
Source: PLoS Genet. 2015 Aug 21;11(8):e1005465. doi: 10.1371/journal.pgen.1005465 (PMC4546604; doi:10.1371/journal.pgen.1005465)
Supplement: S4 Table — (PDF) [file pgen.1005465.s017.pdf]

Motif	Total number of peaks	Number of peak regions with motif	Number of control peak regions with motif	Number of summit regions with motif	Number of flanking regions with motif	Percent of peak regions with motif	Enrichment over control regions	Enrichment at summit over flanking regions	P-value (over control)
Jolma_TEAD4_1	2498	2069	630	1797	982	82.83	3.28	1.83	0
Jolma_TEAD1_1	2498	1931	499	1647	867	77.30	3.87	1.90	0
MA0090_1_TEAD1	2498	1954	814	1666	1015	78.22	2.40	1.64	5.46×10^{-241}
Jolma_TEAD3_1	2498	1740	735	1531	811	69.66	2.37	1.89	1.21×10^{-183}
Jolma_JDP2_1	2498	1072	342	758	537	42.91	3.13	1.41	2.76×10^{-121}
MA0099_2_AP1	2498	1100	424	749	554	44.03	2.59	1.35	6.16×10^{-99}
Jolma_NFE2_1	2498	1119	478	787	592	44.80	2.34	1.33	1.45×10^{-86}
MA0003_1_TFAP2A	2498	888	356	530	570	35.55	2.49	0.93	4.27×10^{-70}
Jolma_TFAP2A_1	2498	912	379	565	600	36.51	2.41	0.94	1.44×10^{-68}
MA0107_1_RELA	2498	1071	544	727	583	42.87	1.97	1.25	1.72×10^{-58}
Jolma_TFAP2B_1	2498	944	441	580	622	37.79	2.14	0.93	2.07×10^{-58}
Jolma_Tcfap2a_1	2498	879	395	539	569	35.19	2.22	0.95	3.29×10^{-57}
Jolma_TFAP2C_1	2498	941	459	580	620	37.67	2.05	0.93	2.23×10^{-53}
Jolma_EBF1_1	2498	935	457	596	576	37.43	2.05	1.03	1.05×10^{-52}
MA0056_1_MZF1	2498	1075	573	615	672	43.03	1.88	0.91	1.18×10^{-52}
MA0144_1_Stat3	2498	1226	728	853	653	49.08	1.68	1.31	4.01×10^{-48}
MA0081_1_SPIB	2498	987	525	622	527	39.51	1.88	1.18	5.72×10^{-47}
MA0154_1_EBF1	2498	1227	747	776	751	49.12	1.64	1.03	1.12×10^{-44}
MA0150_1_NFE2L2	2498	954	517	595	578	38.19	1.84	1.03	7.29×10^{-43}
MA0067_1_Pax2	2498	868	450	555	473	34.75	1.93	1.17	4.46×10^{-42}
MA0105_1_NFKB1	2498	909	488	559	583	36.39	1.86	0.96	3.86×10^{-41}
MA0002_2_RUNX1	2498	1171	717	766	749	46.88	1.63	1.02	8.17×10^{-41}
Jolma_Zic3_1	2498	1041	604	691	712	41.67	1.72	0.97	2.39×10^{-40}
Jolma_Egr1_1	2498	1100	658	703	766	44.03	1.67	0.92	5.95×10^{-40}
Jolma_ZNF740_1	2498	990	573	619	665	39.63	1.73	0.93	6.93×10^{-38}
MA0155_1_INSM1	2498	1170	736	736	784	46.84	1.59	0.94	2.54×10^{-37}
Jolma_ZIC4_1	2498	991	578	646	677	39.67	1.71	0.95	4.04×10^{-37}
Jolma_Zfp740_1	2498	1055	642	665	707	42.23	1.64	0.94	1.10×10^{-35}
Jolma_NFIX_1	2498	892	503	553	589	35.71	1.77	0.94	2.11×10^{-35}
Jolma_ZIC1_1	2498	965	569	615	656	38.63	1.70	0.94	1.09×10^{-34}
Jolma_EGR1_1	2498	839	468	513	558	33.59	1.79	0.92	1.12×10^{-33}
Jolma_NFIB_1	2498	867	512	526	568	34.71	1.69	0.93	5.50×10^{-30}
Jolma_NFIA_1	2498	873	523	537	573	34.95	1.67	0.94	5.49×10^{-29}
Jolma_ELK4_1	2498	566	276	352	305	22.66	2.05	1.15	6.76×10^{-29}
Jolma_EGR4_1	2498	926	570	585	625	37.07	1.62	0.94	9.01×10^{-29}
Jolma_EGR3_1	2498	931	579	585	644	37.27	1.61	0.91	5.00×10^{-28}
Jolma_GLIS2_1	2498	1089	721	706	751	43.59	1.51	0.94	6.45×10^{-28}
Jolma_ELK1_1	2498	622	325	384	341	24.90	1.91	1.13	1.22×10^{-27}
Jolma_RUNX3_1	2498	831	498	543	518	33.27	1.67	1.05	3.38×10^{-27}
MA0061_1_NF-kB	2498	902	559	518	559	36.11	1.61	0.93	3.41×10^{-27}
MA0161_1_NFIC	2498	950	603	587	527	38.03	1.57	1.11	6.94×10^{-27}
MA0101_1_REL	2498	981	636	649	525	39.27	1.54	1.24	4.67×10^{-26}
Jolma_GCM2_1	2498	688	386	442	392	27.54	1.78	1.13	4.72×10^{-26}
MA0014_1_Pax5	2498	862	534	554	600	34.51	1.61	0.92	1.11×10^{-25}
MA0062_2_GABPA	2498	879	549	534	525	35.19	1.60	1.02	1.23×10^{-25}

MA0156_1_FEV	2498	861	536	520	470	34.47	1.61	1.11	3.10×10^{-25}
Jolma_ELF1_1	2498	863	540	562	498	34.55	1.60	1.13	6.92×10^{-25}
Jolma_SP8_1	2498	1017	678	614	694	40.71	1.5	0.88	1.15×10^{-24}
Jolma_ERF_1	2498	676	388	410	399	27.06	1.74	1.03	5.24×10^{-24}
Jolma_RUNX2_1	2498	765	462	481	494	30.62	1.66	0.97	5.50×10^{-24}
Jolma_Egr3_1	2498	762	460	466	494	30.50	1.66	0.94	6.67×10^{-24}
Jolma_Elk3_1	2498	458	223	272	252	18.33	2.05	1.08	4.69×10^{-23}
Jolma_FEV_1	2498	530	278	323	286	21.22	1.91	1.13	6.21×10^{-23}
Jolma_ETV4_1	2498	673	401	410	373	26.94	1.68	1.10	1.81×10^{-21}
Jolma_ETV5_1	2498	289	113	168	159	11.57	2.56	1.06	4.30×10^{-21}
Jolma_KLF13_1	2498	879	584	546	573	35.19	1.50	0.95	1.40×10^{-20}
Jolma_ELF4_1	2498	708	438	430	411	28.34	1.62	1.05	2.77×10^{-20}
Jolma_ETV1_1	2498	449	231	261	249	17.97	1.94	1.05	4.30×10^{-20}
Jolma_ELK3_1	2498	623	371	385	339	24.94	1.68	1.14	1.07×10^{-19}
Jolma_FLI1_1	2498	741	471	451	429	29.66	1.57	1.05	1.43×10^{-19}
Jolma_ERG_1	2498	657	402	398	368	26.30	1.63	1.08	2.88×10^{-19}
Jolma_EGR2_1	2498	563	329	340	339	22.54	1.71	1.00	1.35×10^{-18}
Jolma_GABPA_1	2498	500	282	304	268	20.02	1.77	1.13	4.95×10^{-18}
Jolma_MAX_1	2498	503	285	321	310	20.14	1.76	1.03	6.27×10^{-18}
Jolma_PAX9_1	2498	556	333	330	346	22.26	1.67	0.95	4.30×10^{-17}
Jolma_Mlx_1	2498	271	121	159	149	10.85	2.24	1.07	4.53×10^{-16}
Jolma_NRL_1	2498	588	375	329	352	23.54	1.57	0.93	6.74×10^{-15}
MA0076_1_ELK4	2498	486	296	292	258	19.46	1.64	1.13	4.06×10^{-14}
MA0093_1_USF1	2498	490	304	296	251	19.62	1.61	1.18	1.87×10^{-13}
Jolma_PAX1_1	2498	551	362	346	322	22.06	1.52	1.07	1.52×10^{-12}
Jolma_HINFP1_1	2498	398	237	228	251	15.93	1.68	0.91	2.36×10^{-12}
Jolma_ETV3_1	2498	467	301	270	263	18.69	1.55	1.03	2.48×10^{-11}
Jolma_SPDEF_1	2498	440	287	250	248	17.61	1.53	1.01	2.85×10^{-10}
Jolma_NRF1_1	2498	452	298	277	250	18.09	1.52	1.10	3.69×10^{-10}
Jolma_TP63_1	2498	382	240	217	242	15.29	1.59	0.90	3.84×10^{-10}
Jolma_XBP1_1	2498	261	165	133	154	10.45	1.58	0.86	4.05×10^{-07}
MA0006_1_Arnt_Ahr	2498	308	204	189	142	12.33	1.51	1.33	4.48×10^{-07}
MA0100_1_Myb	2498	220	137	124	115	8.81	1.61	1.08	1.80×10^{-06}
MA0004_1_Arnt	2498	218	136	121	111	8.73	1.60	1.09	2.16×10^{-06}

S4 Table. Known motifs enriched in YAP1/TEAD1 peak regions.
Motifs shown are at least 1.5 fold enriched in YAP1/TEAD1 peak regions compared to control regions and are ranked by P-value (hypergeometric). Colors indicate gene expression levels (FPKM: fragments per kilobase of transcript per million mapped reads) (dark grey FPKM ≥ 10; light grey FPKM ≥ 1; white FPKM < 1).
