# Supplementary material for: YAP1 Exerts Its Transcriptional Control via TEAD-Mediated Activation of Enhancers
Source: PLoS Genet. 2015 Aug 21;11(8):e1005465. doi: 10.1371/journal.pgen.1005465 (PMC4546604; doi:10.1371/journal.pgen.1005465)
Supplement: S5 Table — (PDF) [file pgen.1005465.s018.pdf]

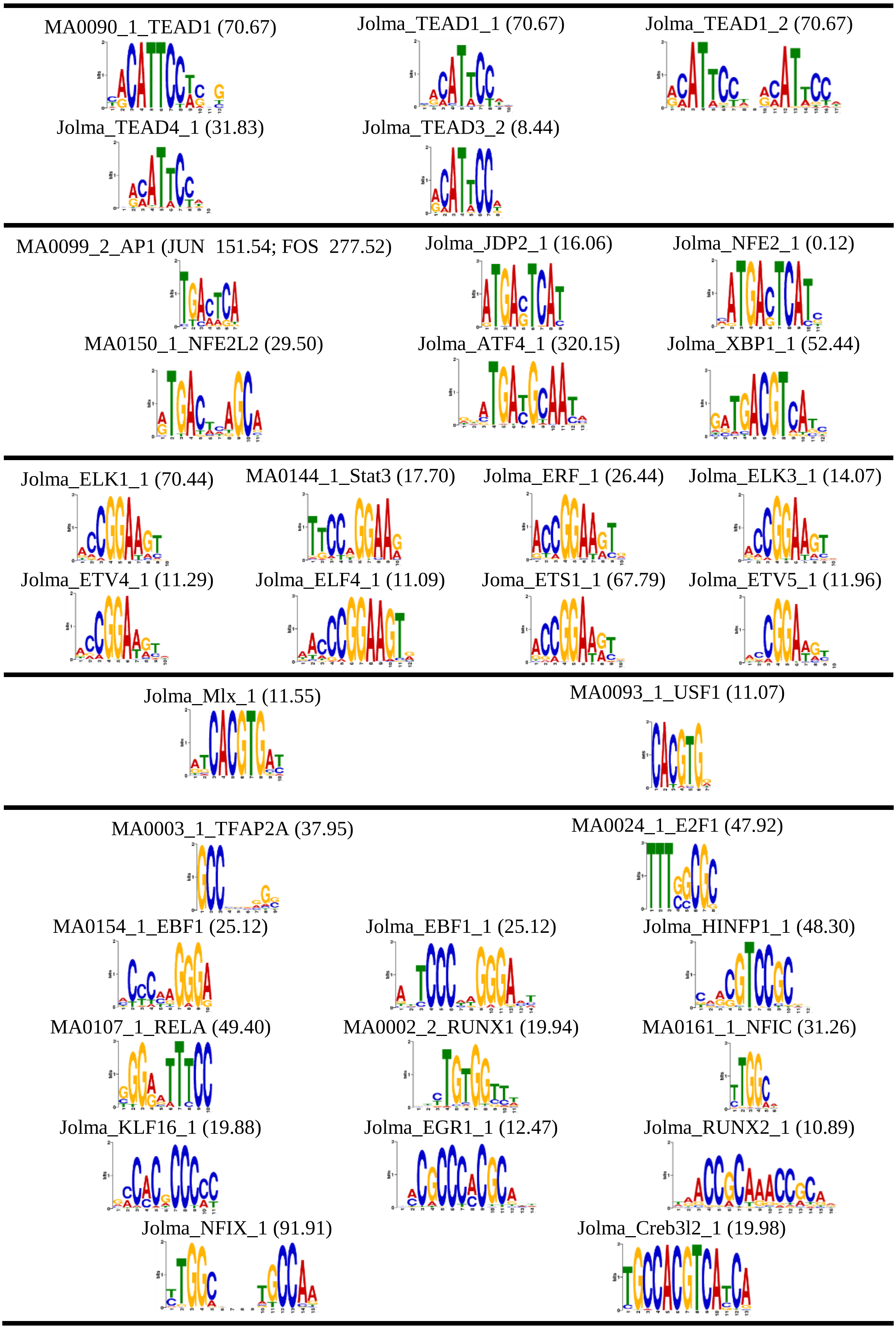

S5 Table. Enriched motif logos.

Logos from motifs in **S4 Table** with corresponding gene expression level (FPKM).
