# Supplementary material for: YAP1 Exerts Its Transcriptional Control via TEAD-Mediated Activation of Enhancers
Source: PLoS Genet. 2015 Aug 21;11(8):e1005465. doi: 10.1371/journal.pgen.1005465 (PMC4546604; doi:10.1371/journal.pgen.1005465)
Supplement: S7 Table — (PDF) [file pgen.1005465.s020.pdf]

ADAMTS5	ADRB2	ANKRD1	ATAD2	BMP4
C4BPB	CDC6	CDK6	CITED2	CTGF
CYR61	DLC1	DUSP4	EFNB2	FGF2
FRMD6	FST	FZD1	GADD45A	GADD45B
GBX2	HAPLN1	HAS2	IGFBP3	INHBA
IRS1	KISS1	LHX4	MAFF	MAMDC2
MOK	NOG	NPPB	PHTF2	PLEKHA7
PLEKHG7	PRICKLE1	PTPRQ	SDPR	SERTAD4
SH2D4A	SHISA9	SLFN12	ST3GAL1	TEAD1
TLL1	TRAM2	TRPC6	VGLL2	VSTM1
ZFP28	ZFP30	ZFP82	ZNF138	ZNF345
ZNF383	ZNF431	ZNF461	ZNF529	ZNF540
ZNF566	ZNF675	ZNF699	ZNF716	ZNF720
ZNF729	ZNF790	ZNF804A	ZNF90	ZNF92

S7 Table. YAP1-target genes in SF268 cells.

List of genes which were down-regulated upon siRNA-mediated depletion of YAP1 and which are associated to YAP1/TEAD1 binding sites in SF268 cells.
